# BriDGE the gap: Improving behavioral research by integrating DAGs and GAMs into experiments

**DOI:** 10.3758/s13428-026-03146-2

**Published:** 2026-09-23

**Authors:** Giuseppe Alessandro Veltri, Sanchayan Banerjee

**Affiliations:** 1 National University of Singapore, Behavioural and Implementation Science Interventions (BISI), Yong Loo Lin School of Medicine, National University of Singapore, Block MD11, Level 2, 10 Medicine Drive, Singapore, 117597 Singapore; 2 Department of Sociology and Social Research, University of Trento, Via Verdi 26, 38122 Trento, Italy; 3 School for Government, King’s College London, Strand, London, WC2R 2LS UK

**Keywords:** Behavioral interventions, Causal inference, Directed acyclic graphs, Generalized additive models, Randomized controlled trials (RCTs)

## Abstract

Understanding the mechanisms through which behavioral interventions work remains a critical challenge in behavioral science. While randomized controlled trials (RCTs) provide reliable evidence for intervention efficacy, they are seldom designed to reveal the underlying causal pathways that drive observed outcomes. We introduce a comprehensive data-driven methodological protocol – BriDGE – that combines advanced causal inference techniques, such as directed acyclic graphs (DAGs), causal discovery algorithms, and generalized additive models (GAMs), to enhance mechanistic insights in behavioral applications. BriDGE modifies conventional experimental analysis with a stepwise approach including DAG-based hypothesis formulation, modeling of nonlinear relationships with GAMs, and detailed mediation analysis. Using bootstrapping and sensitivity checks, BriDGE ensures robust and reliable detection of both direct and indirect effects. We use a simulation study to validate BriDGE’s ability to identify complex causal mechanisms, offering researchers a robust framework for deepening understanding of causal mechanisms and optimizing intervention design. To support adoption, we additionally provide practical guidance on mediator dimensionality, computational feasibility, and simulation-based power planning, including benchmarking templates implemented in the accompanying code. There are natural limitations of BriDGE – we discuss their implications when applied to public policy. We call for a greater integration of these methods in the toolkit of applied policy analysis to bridge the gap from “what works” to “why and how it works”. We also release BriDGE, an open-source R package that implements the workflow to facilitate adoption and reproducibility.

**Supplementary Information:**

The online version contains supplementary material available at https://doi.org/10.3758/s13428-026-03146-2.

## Introduction

Behavioral science interventions are pivotal in addressing a myriad range of societal challenges, from promoting healthy lifestyles to enhancing educational outcomes. Despite these contributions, behavioral science and public policy have reached a level of theoretical and methodological maturity that invites critical reflection (Banerjee & John, 2025). With increased popularity, critiques have emerged from within and outside the field (Hallsworth, 2023; Varazzani & Hubble, 2024), which are crucial for the future development of this field from a research perspective. These critiques mainly center around limitations of individually focused behavioral public policies, such as their limited effectiveness and lack of persistence as found in large meta-analyses (Bakdash & Marusich, 2022; DellaVigna & Linos, 2022; Maier et al., 2022; Mertens et al., 2022), which has led to assertions on the need to move towards more system-based behavioral interventions (Chater & Loewenstein, 2023). We believe this critique against individual-based interventions mainly stems from three fundamental underlying concerns about how we understand behavioral insights: the inability to reveal mechanistic explanations of intervention delivery (Banerjee & Veltri, 2024), the need for a better account of the heterogeneity of behavioral intervention effects, and the consideration of temporal dynamics. In this paper, we turn towards the first issue – mechanistic understanding – which is important for theory development, fine-tuning intervention design, and ensuring effective implementation across diverse contexts (Michie et al., 2011).

Although there is no consensus on the precise definition of a mechanism, common elements include the notion that a mechanism consists of entities and activities organized to produce a particular phenomenon (Illari & Williamson, 2012). These entities and activities can be described at various levels, including mental processes, neurophysiological responses, and social dynamics (Craver, 2006). In the context of behavioral interventions, mechanisms might also involve cognitive processes, emotional responses, social influences, or neurobiological factors that mediate the effect of an intervention on behavior. In the context of behavioral interventions, mechanisms might involve (a) cognitive processes if behavior encompasses cognition, and (b) neural or motor responses if behavior excludes cognition but comprises neural responses that lead to action (e.g., submitting a survey response) (Krpan et al., 2025).

Randomized controlled trials (RCTs), which are considered to be the gold standard for evaluating the efficacy of behavioral interventions, can also be designed to understand mechanisms through which interventions produce change. However, RCTs are seldom designed to assess mechanisms – mainly because efficacy is often prioritized, but also because testing mechanisms requires a more complex design and analytical plan that goes beyond the traditional repertoire of statistical analyses. Traditional statistical methods focus on estimating average treatment effects without disentangling the complex web of mediators and/or moderators that influence treatment effects (Baron & Kenny, 1986). So, despite the high internal validity of RCTs, they often provide limited insights into the causal pathways that mediate the relationship between an intervention and its outcomes, limiting understanding of potential causes of nonlinear relationships, heterogeneity, and adverse or spillover effects. Consequently, interventions may be scaled up without a clear understanding of why or how they work, leading to inconsistent results in different populations or settings (Veltri, 2023).

This excessive enthusiasm for “what works” has long been a persistent issue in behavioral science (Banerjee & John, 2025), largely stemming from the field’s historical development and its close ties to public policy, where cost-effectiveness has been a primary concern. While this focus was shaped by past priorities, it remains a growing challenge today – one that is increasingly difficult to address (Beshears & Kosowsky, 2020). The lack of mechanistic insight hampers the advancement of behavioral theories and the optimization of interventions. It also poses challenges for policymakers and practitioners who need to know not just whether an intervention works, but *how* and *why* it works, to effectively adapt it to new contexts (Grüne-Yanoff et al., 2023; Strassheim, 2021). Addressing this gap requires methodological innovations that can unravel the causal structures underlying observed effects, effectively moving us from a “what works” to a “why and how it works” mindset.

Recent advances in causal inference provide promising tools to enhance mechanistic understanding. Techniques such as directed acyclic graphs (DAGs) offer rigorous frameworks for modeling and testing causal relationships beyond mere associations (Pearl, 2009; Spirtes et al., 2001). However, as previous research has highlighted (Bailey et al., 2024), causal inference in human behavior faces additional challenges, including effect heterogeneity, confounding, and the complexity of human interactions. Thus, combining multiple approaches and triangulating causal estimates from experimental and observational data is essential for drawing robust inferences.

In this article, we propose a novel methodological protocol – BriDGE – that leverages advanced causal inference techniques to provide a more nuanced analysis of RCTs in behavioral science. BriDGE – metaphorically relating to bridging the gap from “what works” to “why and how it works” – aims to improve *B*ehavioral *r*esearch by *i*ntegrating *D*AGs, causal discovery algorithms, and mediation analysis using *G*eneralized additive models (GAMs) in *E*xperiments. By combining these techniques, BriDGE offers a powerful approach to model complex causal mechanisms involving nonlinear relationships and interactions, moving beyond average treatment effects to develop a comprehensive understanding of the causal pathways in behavioral interventions. BriDGE aligns with recent calls for more integrative experimental designs in the social and behavioral sciences (Almaatouq et al., 2024), which emphasize the importance of exploring a comprehensive design space to uncover complex interactions and contextual factors. Moreover, the integration of observational data, alongside experimental data, enhances the robustness of causal inferences, especially in cases where RCTs face challenges of under- or over-control of variables (Bailey et al., 2024). BriDGE enables researchers to carefully consider both forward and reverse causal questions to refine their causal models, ensuring that both the direct and indirect effects of interventions are understood and well identified.

## Going beyond RCTs

### Formalizing behavioral mechanisms using causal models

Understanding mechanisms underlying relationships that behavioral interventions intend to modify requires a formal framework that is capable of representing complex causal relationships. Causal models, such as the potential outcomes framework – also known as the Neyman–Rubin–Holland model (Rubin, 1990) – provide such a foundation, enabling researchers to conceptualize and analyze intervention pathways. However, these standard causal models focus mostly on establishing effectiveness rather than explicitly studying mechanisms underlying behaviors interventions.

One useful tool to understand mechanisms is the directed acyclic graph (DAG), which is a graphical representation of hypothesized causal relationships between variables (Pearl, 1995). In a DAG, nodes represent variables – such as cognitive processes, emotional states, or social influences – while directed edges indicate causal influences (Morgan & Winship, 2015). The acyclic structure ensures unidirectional, time-ordered relationships, preventing feedback loops that complicate causal inference. Moderation and mediation models typically focus on a small set of focal variables (e.g., X → M → Y), rely on statistical frameworks like regression or SEM, and emphasize whether effects differ by subgroup (moderation) or flow through a mediator (mediation). In contrast, DAGs explicitly encode hypothesized causal directions among multiple variables, highlight potential confounders and colliders, and show which variables must (or must not) be adjusted for to identify causal effects. They are conceptual tools that reveal broader causal structures, whereas moderation/mediation models generally test specific theoretical pathways without formally addressing all sources of confounding. Thus, DAGs offer a more comprehensive guide to causal inference, while moderation/mediation models center on particular direct or indirect relationships.

DAGs make explicit the assumptions about how interventions influence mediators and outcomes, helping to identify potential confounders, mediators, and moderators. This graphical approach supports both study design and data analysis, formalizing the entities (variables) and interactions (causal mechanisms) involved in behavioral interventions (Craver, 2006; Illari & Williamson, 2012). However, as noted by Bailey et al. (2024), DAGs alone may not fully resolve challenges like confounding or interference in social settings. Researchers should triangulate causal inferences by incorporating observational studies or quasi-experimental designs, which provide complementary evidence. This triangulation is crucial for addressing biases such as under- or over-control, common in experimental studies. Additionally, behavioral mechanisms often involve nonlinear relationships that linear models struggle to capture.

Generalized additive models (GAMs) offer a flexible alternative by incorporating smooth functions to model nonlinear effects and interactions (Hastie & Tibshirani, 1990). In mechanistic testing, GAMs refine DAG-based insights by estimating functional relationships between variables. For instance, a mediator may exhibit a threshold effect on the outcome, or its influence may increase at a diminishing rate. By leveraging GAMs, researchers can better quantify these effects, improving mechanistic understanding. In practice, we implement GAMs in mgcv (Wood, 2011, 2017).

As Bailey et al. (2024) highlights, interventions often affect multiple variables simultaneously (“fat-handed” interventions), requiring models that accommodate complexity. GAMs provide this flexibility without imposing rigid parametric assumptions, making them particularly valuable in behavioral science research.

Building on DAGs and GAMs, our protocol underscores the need to integrate multiple sources of evidence to uncover robust causal mechanisms. Given challenges such as effect heterogeneity, confounding, and intervention timing, researchers should triangulate findings from RCTs, quasi-experiments, and observational studies. This ensures that causal inferences remain robust across different data sources and analytical methods.

### Data collection for mechanistic testing

Randomized controlled trials (RCTs) are widely regarded as the gold standard for evaluating the efficacy of behavioral interventions. Traditional RCTs often fail to understand the mechanisms of behavior change, which requires a study design that explicitly captures causal pathways. RCTs must, therefore, go beyond assessing overall efficacy and systematically measure mediators and moderators along the hypothesized causal pathway. Nonetheless, this entails incorporating measures of cognitive, emotional, or behavioral variables that intervene between the intervention and the outcome. However, reliably capturing these variables presents significant challenges. Measurements must be precise, validated, and sensitive to change, yet RCTs often lack the granularity needed to detect subtle mechanistic effects.

Additionally, mechanistic analyses informed by DAGs typically require larger sample sizes than standard efficacy analyses, as causal pathways involving indirect or subtle effects often demand greater statistical power. Consequently, power calculations must explicitly consider these causal relationships, yet many RCTs remain underpowered for reliably identifying mechanisms. Further, attrition over time can compromise the integrity of mediation analyses (Imai et al., 2010; Tingley et al., 2014), particularly in longitudinal studies, where missing data complicates causal inference. While statistical techniques like multiple imputation can help, they do not fully resolve the limitations inherent in RCT designs.

Even with high-quality data collection – ensuring standardized procedures, rigorous training, and ongoing monitoring – RCTs still face structural constraints in mechanism testing. Ethical concerns also arise when measuring mediators, especially if they involve sensitive psychological or behavioral data. Balancing comprehensive data collection with participant burden is crucial, but this trade-off often limits the depth of mechanistic insights.

Rather than relying solely on RCTs to infer mechanisms, alternative protocols such as directed acyclic graphs (DAGs) and generalized additive models (GAMs) provide more flexible and informative approaches. DAGs explicitly represent hypothesized causal relationships, helping researchers clarify assumptions, identify confounders, and improve study design. GAMs, in turn, allow for modeling nonlinear and interactive effects that traditional mediation analyses within RCTs may overlook. Alternatively, they can be used to analyze RCT data from a mechanistic angle that has already been collected.

As highlighted by Almaatouq et al. (2024), an integrative experiment design – one that systematically samples from a broader design space – offers a more effective way to capture the complexity of causal mechanisms. By moving beyond conventional RCT frameworks and embracing tools like DAGs and GAMs, researchers can develop a more nuanced understanding of how behavioral interventions drive change.

**Fig. 1:**
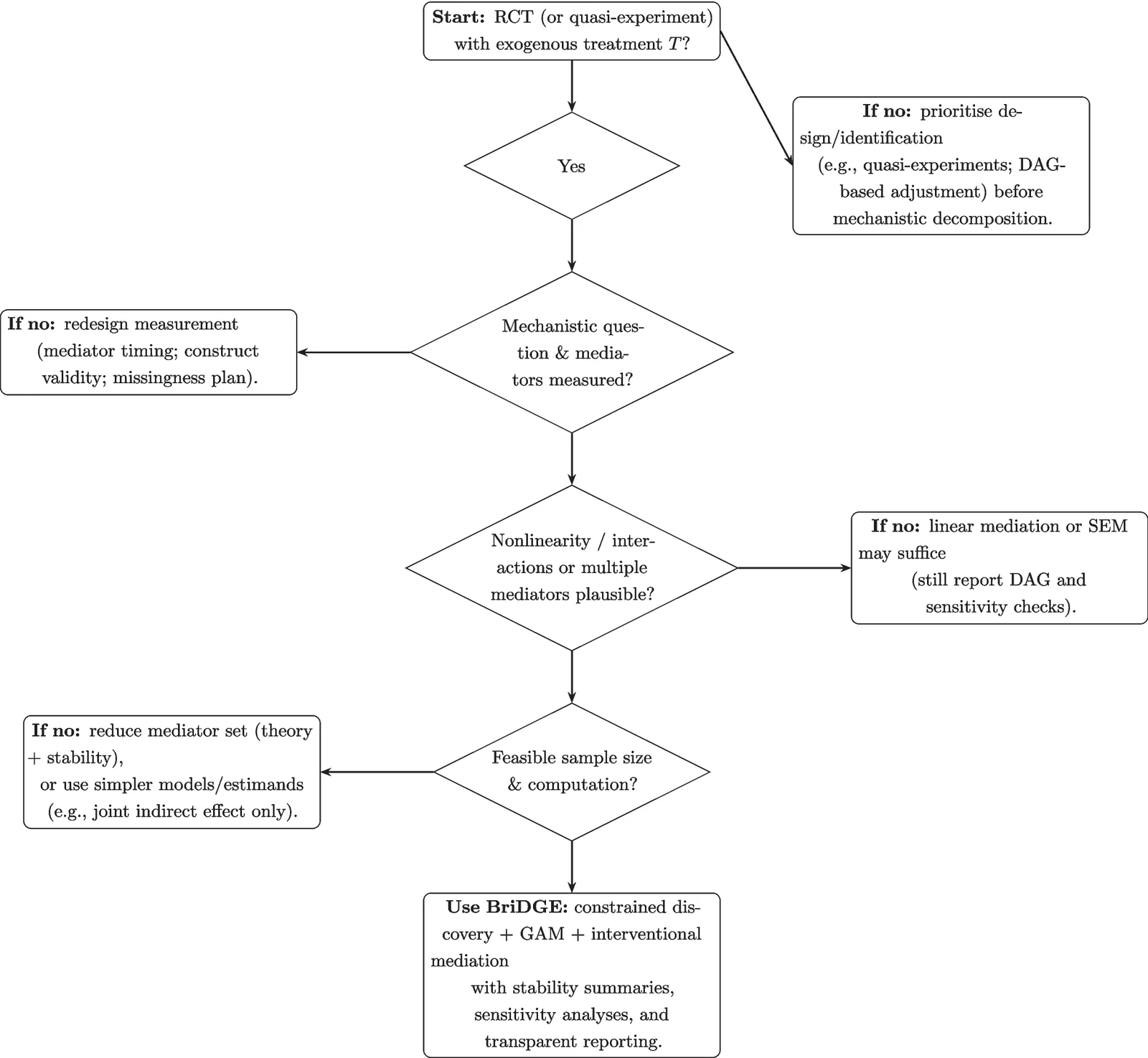
Decision flowchart for selecting BriDGE versus simpler alternatives. The “feasibility” node refers to both statistical feasibility (power for mediation estimands; stability of causal discovery) and computational feasibility (runtime as a function of *N*, mediator dimensionality, and bootstrap size)

## BriDGE – Improving behavioral research by integrating DAGs and GAMs in experiments

Building upon the theoretical foundations of DAGs and GAMs and the limitations of data collection strategies in conventional RCTs as outlined above, we propose a comprehensive methodological protocol – “BriDGE” – to improve current behavioral research by integrating DAGs and GAMs in experimental methods. BriDGE, as the name suggests, is designed to bridge the gap in applied behavioral science so that we can uncover and analyze the mechanisms underlying behavioral interventions. This protocol integrates causal models, advanced statistical techniques, and rigorous analysis steps to provide a systematic approach to mechanistic testing.

The unique contribution of this protocol is its ability to synergistically integrate multiple advanced causal inference techniques to address the complexities of behavioral data. By combining DAGs, causal discovery algorithms, and GAMs, the protocol provides a robust framework capable of handling nonlinear relationships, interactions, and high-dimensional data. This integrated approach goes beyond traditional methods by explicitly modeling the causal structure and functional forms of relationships, thereby offering practical advantages for researchers and practitioners seeking to optimize interventions based on mechanistic insights. This protocol takes the following steps:

- Step 1: Construct a directed acyclic graph (DAG) that represents the hypothesized causal relationships among the intervention, mediators, and outcomes. This graphical model serves as a blueprint for the analysis, making explicit the assumed mechanisms and guiding the selection of variables for measurement.
- Step 2: Collect data, according to the considerations specific to RCTs in behavioral science, ensuring that mediators and outcomes are measured at appropriate times and with sufficient precision (see James and Brett (1984)). This data collection feeds directly into the subsequent analytical steps.
- Step 3: Apply causal discovery algorithms (Spirtes et al. (2001); Colombo et al. (2012)) to empirically interrogate the hypothesized DAG *and quantify structural uncertainty*. We leverage RCT constraints (e.g., treatment exogeneity via blacklists/whitelists) and implement MMHC via *bnlearn* (Scutari, 2010) with mutual-information tests and the package’s default scoring for structure learning. Crucially, we accompany the point-estimate discovered DAG with bootstrap arc-strength (stability) summaries and treat low-stability edges as *ambiguous hypotheses* rather than definitive causal claims. Because discretization choices can influence mutual-information tests, we recommend reporting sensitivity to the number of bins and (when applicable) to alternative conditional-independence tests.
- Step 4: Employ generalized additive models (GAMs) to model the relationships between variables, capturing nonlinear effects and interactions that are often present in behavioral data. This step enhances the accuracy of effect estimates and provides deeper insights into the functional forms of the relationships among the intervention, mediators, and outcomes. We implement GAMs using mgcv (Wood, 2011, 2017).
- Step 5: Conduct mediation analysis to decompose the total effect of the intervention into direct and indirect effects through identified mediators. While the DAG phase helps figure out which causal routes exist, the mediation analysis quantifies how much of the treatment effect flows through each identified route. By quantifying these effects, the analysis reveals the pathways through which the intervention operates, offering a detailed understanding of the mechanisms at play.
- Step 6: Quantify uncertainty and robustness using nonparametric bootstrapping (including *bias-corrected and accelerated* confidence intervals as default), and targeted sensitivity analyses addressing (i) discretization and discovery settings, (ii) GAM basis size/specification, and (iii) mediator measurement error and potential mediator–outcome confounding. For BCa intervals, we recommend at least B≥1000 bootstrap replicates (we use B=2000 in the simulation), and we provide benchmarking templates (runtime by *N* and mediator dimensionality) to guide feasibility decisions.

### Implementation note

All steps of BriDGE are implemented in the R package bridgeR, which provides functions for (i) DAG specification and validation, (ii) constrained causal discovery (MMHC with whitelists/blacklists), (iii) GAM fitting with nonlinear, and (iv) mediation with bootstrap uncertainty. The package enables end-to-end replication of the simulation and templates for new studies (https://doi.org/10.17605/OSF.IO/NSMWB).

BriDGE effectively addresses challenges such as unmeasured confounding and high-dimensional data by utilizing causal discovery algorithms to uncover hidden relationships and GAMs to model complex patterns. For researchers and practitioners, the protocol offers practical benefits, including enhanced accuracy in effect estimation, the ability to tailor interventions based on detailed causal insights, and the potential to improve intervention effectiveness by understanding the underlying mechanisms.

### Decision guidance: When to apply BriDGE versus simpler approaches

To support applied use, Fig. 1 provides a pragmatic decision flowchart for choosing between (i) BriDGE, (ii) simpler single-mediator or linear mediation models, and (iii) designs that prioritize additional measurement or temporal separation. The goal is not to replace domain theory, but to provide a transparent rubric for matching analytic complexity to the scientific question, the mediator set, and the data quality. Because BriDGE is explicitly mechanism-oriented, several experimental-design choices materially affect what can be learned before any modeling step is run. In particular, credibility of mechanistic claims is strengthened by (i) temporal separation between mediators and outcomes when feasible, (ii) measurement strategies that reduce common-method bias (e.g., combining self-report mediators with behavioral or administrative outcomes or multi-method mediator measurement), (iii) baseline measurement of key mediators/outcomes to support adjustment and to distinguish change from level, and (iv) a pre-specified rationale for mediator inclusion grounded in construct validity and expected timing. We therefore intend Fig. 1 to function not only as an analysis decision aid but also as a compact checklist for mechanism-oriented study design.

## BriDGE in practice: Simulation study and semi-synthetic benchmark

This section illustrates BriDGE via two complementary evaluation designs. First, we use a fully synthetic simulation in which the causal graph and all target interventional effects are known, allowing clean validation under controlled departures from linearity and additivity. Second, we use a semi-synthetic benchmark built on an RCT covariate scaffold (JOBS II), which preserves realistic covariate support and mixed measurement scales while retaining a known ground truth for the post-treatment mechanism. We describe both designs here and report their empirical performance in Section “Results: simulation study and semi-synthetic benchmark”.

### Data generation

The dataset was generated with a sample size of 1000 units. The treatment (T) was randomly assigned as a binary variable. The two mediators ($M_1$ and $M_2$) and the outcome (Y) were generated using nonlinear functions of the treatment, as well as random noise. The specific relationships are expressed mathematically as follows:

*(1) Treatment Assignment*

$$ T_i \sim \textrm{Bernoulli}(0.5) $$

*(2) Mediator* $$M_{1i}$$

$$ M_{1i} \;=\; 1.0 \,\cdot \, T_i \;+\; \epsilon _{1i} \;+\; \alpha \,\sin \bigl (\nu _{1i}\bigr ) $$

*(3) Mediator* $$M_{2i}$$

$$ M_{2i} \;=\; 0.8 \,\cdot \, T_i \;+\; \epsilon _{2i} \;+\; \alpha \,\exp \!\Bigl (\tfrac{\nu _{2i}}{2}\Bigr ) $$

*(4) Outcome* $$Y_i$$

$$ \begin{aligned} Y_i \;\!=\;&\; 1.5\,T_i \;\!+\; 1.2\,M_{1i} \;\!+\; 0.9\,M_{2i} \;+\; 0.5\,M_{1i}^2 \;+\; 0.3\,M_{1i}\,M_{2i} \\&\quad +\; 0.5 \,\bigl (T_i \times M_{1i}\bigr ) \;+\; 0.7 \,\bigl (T_i \times M_{2i}\bigr ) \;+\; \epsilon _{3i}. \end{aligned} $$

*Explanation:*

- *Treatment* $T_i$ *:* Each unit *i* is randomly assigned to treatment ($T_i = 1$) or control ($T_i = 0$) with probability 0.5. Hence,
  $$ T_i \sim \textrm{Bernoulli}(0.5). $$
- *Mediator* $M_{1i}$ *:* Defined as a linear function of $T_i$ with coefficient 1.0, plus random noise $\epsilon _{1i}\sim \mathcal {N}(0,1)$, and a sine-based nonlinear term $\alpha \,\sin (\nu _{1i})$ where $\nu _{1i}\sim \mathcal {N}(0,1)$. The parameter α (a “nonlinear strength” factor) adjusts the amplitude of the sine function.
- **Mediator** $M_{2i}$ **:** Has a coefficient 0.8 for $T_i$, noise $\epsilon _{2i}\sim \mathcal {N}(0,1)$, and an exponential nonlinear term $\alpha \,\exp \!\bigl (\nu _{2i}/2\bigr )$, where $\nu _{2i}\sim \mathcal {N}(0,1)$.
- **Outcome** $$Y_i$$ **:** Includes:
  - A direct effect of treatment: $1.5\,T_i$.
  - Linear effects of the mediators: $1.2\,M_{1i}$ and $0.9\,M_{2i}$.
  - A quadratic term $$0.5\,M_{1i}^2$$ and an interaction $$0.3\,M_{1i}$$$$\,M_{2i}$$.
  - Treatment-by-mediator interaction terms: $0.5\,(T_i \times M_{1i})$ and $0.7\,(T_i \times M_{2i})$.
  - Independent noise $\epsilon _{3i}\sim \mathcal {N}(0,1)$ added to the outcome.

#### Reproducibility

Unless otherwise noted, we set the sample size to N=1000, the nonlinearity parameter to α=0.5, and fix the random seed at 42 for data generation.

**Fig. 2:**
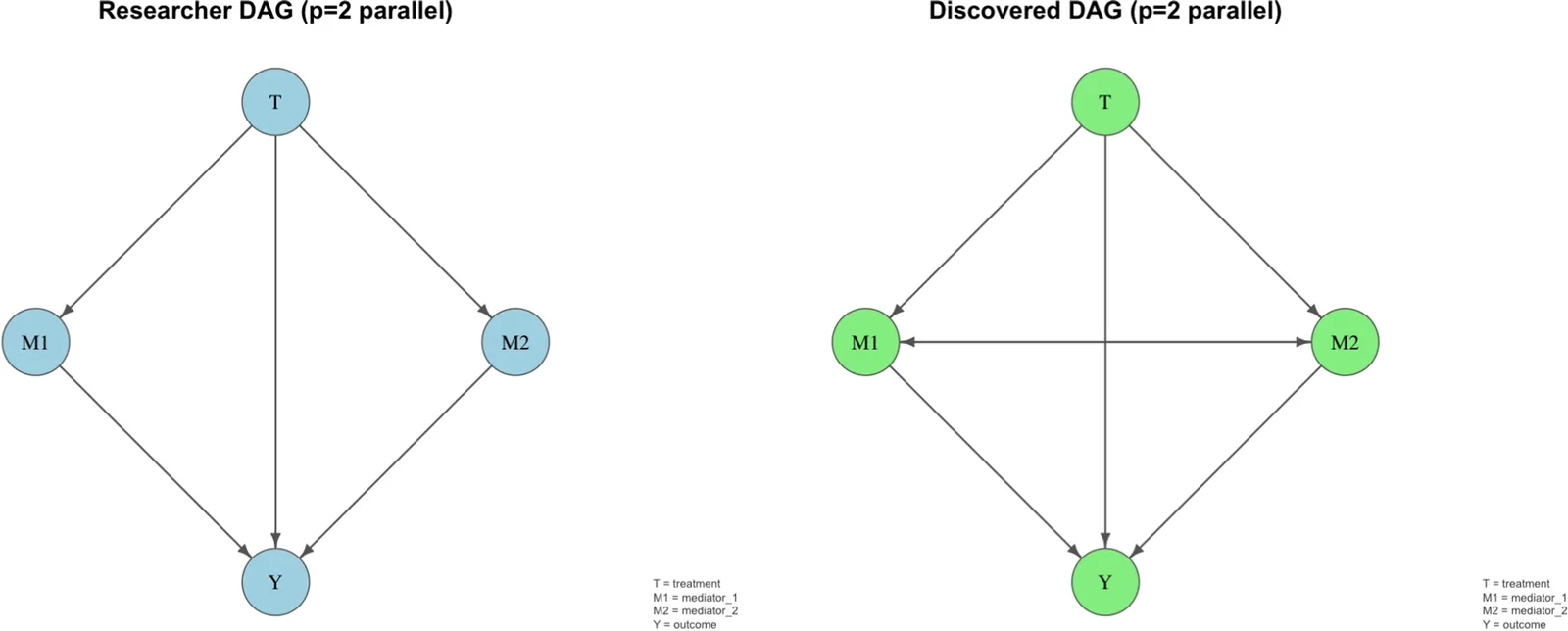
Baseline two-mediator scenario (p=2). *Left*: Researcher’s assumed DAG. *Right*: Discovered DAG (MMHC with treatment$\rightarrow \{M_1, M_2\}$ whitelisted and inbound edges to *T* blacklisted). Node labels are abbreviated: T = treatment, M1/M2 = mediators, Y = outcome

#### Extended simulation scenarios for mediator dimensionality

To address scalability beyond the two-mediator setting, we additionally implement simulation families with $p\in \{3,5\}$ mediators, including (i) a *sequential* (chain) mediation structure and (ii) a *mixed* structure containing both true and null mediators. For each scenario, we report (a) DAG recovery metrics (SHD, precision/recall, and bootstrap arc-strength stability), (b) interventional mediation estimands (direct effect and joint/marginal interventional indirect effects), and (c) computational scaling as a function of *N*, *p*, and bootstrap size. These results are produced by the companion simulation script and are summarized in Section “Practical guidance for applied use and feasibility” (with full tables/figures suitable for Online Supplementary Material). Finally, to illustrate how realistic mediator measurement error can induce theory–data conflicts in causal discovery, we include a mediator measurement-error stress test in which $M_2$ is perturbed prior to discovery.

### Causal directed acyclic graph (DAG)

The causal assumptions of the study were formalized using a directed acyclic graph (DAG), which depicts the treatment as the parent of both mediators and the outcome (Fig. 2). We first construct a researcher-specified DAG based on theoretical assumptions about the intervention’s causal mechanisms, encoding expected relationships between treatment, mediators, and the outcome. However, theoretical models may not always capture the true data-generating process. To test whether our assumed causal structure aligns with the empirical data, we apply a causal discovery algorithm (MMHC) to infer a discovered DAG while preserving experimental constraints (e.g., respecting randomization). By comparing the researcher’s DAG with the discovered DAG, we assess whether the assumed causal pathways are empirically supported or whether alternative mechanisms emerge (Fig. 2).

To complement our theoretical assumptions about the causal structure, we employ a *causal discovery* algorithm to learn a “discovered DAG” directly from the data. Specifically, we apply the MMHC (Max-Min Hill-Climbing) algorithm from the bnlearn package (Scutari, 2010), which combines both constraint-based and score-based searches.

Before running MMHC, we discretize continuous variables so that we can use mutual information tests more effectively. We also incorporate prior knowledge by:

1. *Whitelisting* edges from the treatment variable to the mediators, reflecting the experimental fact that treatment is randomized and thus influences each mediator.
2. *Blacklisting* any edges into the treatment variable, ensuring no backward edges from mediators or outcomes into the treatment. This preserves the logic of random assignment.

These constraints allow the discovered DAG to remain consistent with key experimental design facts while still inferring other relationships – particularly among the mediators and the outcome. Once the discovery process is complete, we compare the learned DAG to our proposed *researcher’s DAG* to evaluate how well our initial theoretical assumptions align with the data-driven structure.

#### Discovery settings

Unless indicated otherwise, we discretize continuous variables using equal-frequency *quintiles* (g=5) and apply mutual-information tests at α=0.05. We whitelist edges from the treatment to the mediators, $T \rightarrow \{M_1, M_2\}$, and blacklist any inbound edges to *T*. To address the arbitrariness of discretization and test-level choices, we additionally report a discovery sensitivity grid varying $g\in \{3,4,5,6\}$ and $\alpha \in \{0.01,0.05,0.10\}$, summarizing (i) structural Hamming distance and directed-edge overlap versus the researcher DAG, and (ii) arc-strength stability under bootstrapped discovery.

DAG comparison figures for the extended mediator scenarios (p=3 sequential chain and p=5 mixed) are provided in the Technical Annex (Annex Figs. A4–A5).

### Generalized additive models (GAMs)

Generalized additive models (GAMs) were employed to flexibly model nonlinear mediator–outcome relationships and empirically accommodate interactions suggested by either theory (researcher DAG) or the discovery stage. The key modeling choice is to retain the exogenous treatment assignment (and its main effect) while allowing smooth, data-adaptive functions of mediators.

#### Mediator models

Because *T* is binary and randomized, we model each mediator with a parsimonious linear regression on *T*:

$$ M_j \,=\, \beta _{0,M_j} + \beta _{T,M_j}\,T + \varepsilon _{M_j},\qquad j\in \{1,2\}. $$

#### Outcome model

In the baseline two-mediator simulation, the data-generating process includes (i) nonlinear main effects of mediators, (ii) mediator–mediator interaction, and (iii) treatment–mediator effect modification (Eq. 4). To capture these features, we fit the following GAM:

$$\begin{aligned} Y \,=\,&\beta _{0,Y} + \beta _{T,Y}\,T + s_1(M_1) + s_2(M_2) + t\!i(M_1, M_2) \\&\quad + s_1(M_1,\,\text {by}=T) + s_2(M_2,\,\text {by}=T) + \varepsilon _Y, \end{aligned}$$

where $s_j(\cdot )$ are thin-plate regression splines and $t\!i(\cdot ,\cdot )$ denotes a tensor-product interaction smooth. The “by” smooths allow treatment-specific deviations in the mediator effects (effect modification), while keeping a single, interpretable main effect of *T*. In higher-dimensional mediator settings, we recommend restricting interaction smooths to theoretically motivated pairs or to pairs supported by stable edges in the discovered DAG (Section “Practical guidance for applied use and feasibility”).

#### Clarifying interaction structure (Eq. 4)

The presence of an $M_1\times M_2$ interaction term and separate $T\times M_1$ and $T\times M_2$ interaction terms does *not* imply a three-way interaction $T\times M_1\times M_2$; a three-way effect would require an explicit term (e.g., $t\!i(M_1,M_2,\,\text {by}=T)$ in a GAM). We therefore treat three-way interactions as optional, to be included only when suggested by substantive theory or clearly supported by the data (e.g., stable evidence for treatment-specific mediator–mediator synergy).

#### Implementation details

GAMs are fit in R using *mgcv* Wood (2011, 2017) with a Gaussian family (identity link), thin-plate regression splines, smoothing parameters estimated via REML, and default basis dimensions (e.g., k=10) subject to diagnostic checks (e.g., gam.check).

The outcome GAM captures the functional forms implied by the data-generating process. As is standard for GAM diagnostics, each curve or surface represents a conditional partial effect on the outcome scale, with other predictors held at reference values.

- *Nonlinear main effects:* The $s_1(M_1)$ smooth shows pronounced curvature, consistent with the quadratic component in the data-generating process, whereas $s_2(M_2)$ is closer to monotone.
- *Mediator synergy:* The $t\!i(M_1,M_2)$ surface (when included) visualizes departures from additivity. Regions where the surface is positive indicate joint mediator configurations that increase *Y* more than expected under purely additive mediator effects.
- *Effect modification:* The treatment-specific deviation smooths $s_j(M_j,\,\text {by}=T)$ (when included) provide a direct visual representation of $T\times M_j$ interactions: they quantify how the mediator effect differs in the treated group relative to control.

### Mediation and bootstrapping

Mediation effects are estimated via Monte Carlo *g*-computa-tion using the fitted mediator and outcome models. We report *interventional* estimands that remain well defined with multiple mediators and treatment–mediator interactions, and quantify uncertainty using a nonparametric bootstrap with *bias-corrected and accelerated* (BCa) confidence intervals.

#### Bootstrap inference

Mediation estimands frequently yield asymmetric and biased bootstrap sampling distributions (especially under nonlinear outcome models). We therefore use BCa intervals rather than standard percentile intervals. For numerical stability, we use B=2000 bootstrap replicates in the simulation (and recommend B≥1000 as a practical minimum), with optional parallelization implemented in bridgeR.

#### Estimands

For *p* mediators, we focus on: (i) the interventional direct effect (IDE), (ii) the joint interventional indirect effect (JIIE), and (iii) marginal interventional indirect effects that isolate the intervention on each mediator distribution while holding the remaining mediators at their control distribution. Because mediator–mediator interactions imply that marginal indirect effects need not sum to the total effect, we treat the JIIE as the primary indirect summary and report marginal effects as complementary diagnostics (Table 1).

**Table 1:** Interventional mediation estimands for the baseline two-mediator simulation (p=2; point estimates with 95% BCa confidence intervals)

Effect	Estimate	CI_lo_pct	CI_hi_pct	CI_lo_bca	CI_hi_bca
ide	2.074	1.932	2.248	1.923	2.184
jiie	3.495	3.318	3.938	3.305	3.653
te	7.026	6.764	7.521	6.736	7.340
iie_mediator_1	1.818	1.657	2.056	1.649	2.050
iie_mediator_2	1.050	0.957	1.170	0.967	1.176

Full details of the estimation strategy – including Monte Carlo integration, bootstrap implementation, definitions of all estimands, and discovery-stage stability analyses—are provided in the Technical Annex (including Annex Tables A2 and A8–A14).

### Semi-synthetic benchmark on an RCT scaffold (JOBS II)

To complement the fully synthetic simulations (where the full data-generating process is known by design) with a benchmark that reflects realistic covariate distributions, we conducted a *semi-synthetic* evaluation using the JOBS II randomized field experiment dataset as a scaffold (as distributed in the R mediation package). Specifically, we retained the observed randomized treatment assignment and baseline covariates from JOBS II, but generated two post-treatment mediators and the outcome from a known structural model that mirrors the non-additive features emphasized in BriDGE (nonlinear mediator effects, treatment–mediator effect modification, and mediator–mediator interaction in the outcome model). This construction preserves realistic support, scaling, and dependence patterns in baseline measures while maintaining a known ground truth for both the causal graph and the target interventional mediation estimands.

Fully synthetic simulations enable definitive validation but can under-represent the distributional irregularities that commonly dominate behavioral datasets (e.g., skewness, heaping/mass points, and strong baseline correlations). The semi-synthetic JOBS II scaffold retains these empirical features while keeping the post-treatment mechanism – and therefore the target interventional effects – known. This provides an intermediate evaluation tier between purely synthetic simulation and fully empirical case studies.

The benchmark isolates two practical questions that applied users face when deploying BriDGE: (i) whether constrained causal discovery remains stable when variables have realistic support and mixed measurement scales, and (ii) whether the mediation decomposition recovers known mechanistic effects under correct specification and how it changes under plausible misspecification. To make (ii) explicit, we estimate effects under (a) a primary interaction-capable outcome GAM aligned with the benchmark DGP and (b) an additive-only outcome GAM treated as a deliberate misspecification. Because multi-mediator non-additive settings make mediator-specific attribution inherently sensitive to how the joint mediator distribution is perturbed, we emphasize the interventional direct effect (IDE), the joint interventional indirect effect (JIIE), and the total effect (TE) as the primary mechanistic summaries.

In this benchmark, the interventional direct effect (IDE) can be read as the portion of the treatment effect that remains when the mediator distribution is held at its control regime, whereas the joint interventional indirect effect (JIIE) summarizes how much the outcome changes when the *entire joint* mediator distribution shifts from its control to its treated regime while holding treatment fixed. The total effect (TE) is the overall difference between treatment and control. Because the benchmark ground truth is known, agreement between estimated and true IDE/JIIE/TE provides an empirical check that the end-to-end pipeline (constrained discovery, GAM specification, and Monte Carlo *g*-computation) is functioning as intended. Conversely, disagreement under the additive-only specification illustrates how mechanistic conclusions can be distorted when non-additive structure is omitted.

**Fig. 3:**
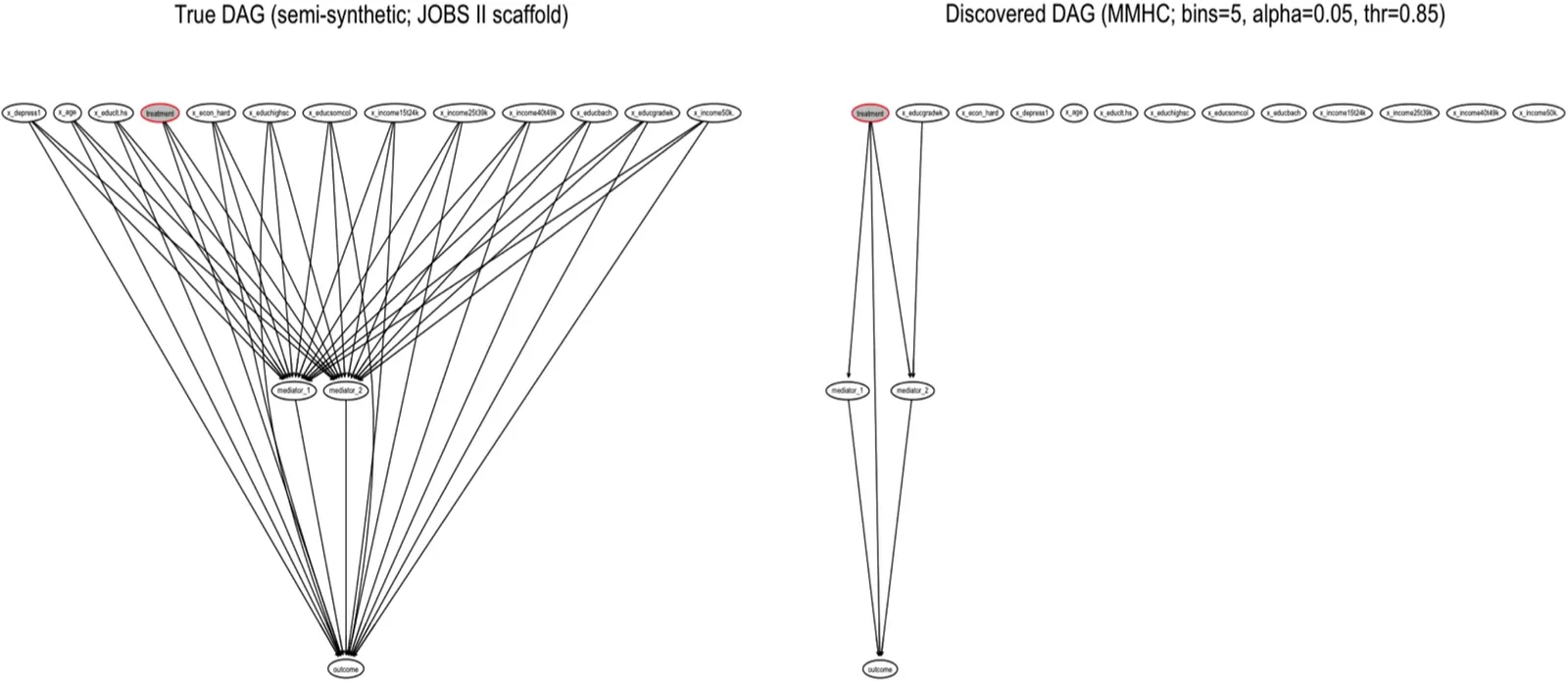
Semi-synthetic JOBS II scaffold: true DAG (*left*) and discovered averaged DAG (*right*) under constrained MMHC and stability filtering

We report the semi-synthetic benchmark results *after* the synthetic simulation results in Section “Semi-synthetic benchmark results (JOBS II scaffold)” (see Fig. 3 and Table 4). The benchmark should be interpreted as a methodological validation exercise rather than as a substantive reanalysis of the JOBS II intervention.

## Results: simulation study and semi-synthetic benchmark

We first summarize results for the fully synthetic simulation scenarios (discovery, mediation, and sensitivity analyses). We then report the semi-synthetic benchmark results on the JOBS II scaffold (Section “Semi-synthetic benchmark results (JOBS II scaffold)”), which serves as a realism check for the BriDGE pipeline under empirical covariate distributions.

### Causal discovery and mediation results

We compare the researcher-specified DAG and the discovered DAG using directed-edge overlap, and we quantify discovery uncertainty via bootstrap arc-strength stability. This directly addresses the practical concern that, in finite samples and under discretization-based conditional-independence testing, edge orientations may be weakly identified. Table 2 summarizes the principal discrepancies (if any) alongside arc-strength and directionality summaries (auto-generated by the companion script).

**Table 2:** Researcher DAG versus discovered DAG (p=2): edge discrepancies (auto-generated)

from	to	Researcher	Discovered
mediator_1	mediator_2	FALSE	TRUE
mediator_1	outcome	TRUE	TRUE
mediator_2	mediator_1	FALSE	TRUE
mediator_2	outcome	TRUE	TRUE
treatment	mediator_1	TRUE	TRUE
treatment	mediator_2	TRUE	TRUE
treatment	outcome	TRUE	TRUE

Extended DAG comparison tables for the p=3 and p=5 scenarios are provided in the Technical Annex (Annex Tables A9–A10).

Where the discovery output conflicts with the researcher DAG, we treat the discrepancy as a hypothesis-generation step and apply the concrete resolution workflow in Section “Resolving discrepancies between proposed and discovered causal models” (temporal constraints, stability thresholds, alternative discovery algorithms, and sensitivity analyses). In the baseline simulation, the only substantive discrepancy concerns the $M_2$–*Y* orientation; bootstrap stability summaries indicate whether this orientation is reliably supported or weakly identified under discretization and finite-sample noise.

Because MMHC, HC, and TABU converge on the same structure in the baseline simulation, the baseline triangulation is best interpreted as evidence that this particular simulated setting is not algorithmically fragile. To make the discrepancy-resolution workflow more diagnostic, the Technical Annex now adds an algorithm-divergence stress test (Annex Table A7) in which the mediator–outcome signal is weakened, and mediator measurement noise is increased; in that setting, algorithm-specific edge orientations are treated as ambiguous hypotheses rather than definitive causal claims.

#### Comparative analysis

Table 3 summarizes mean differences in the mediators and outcome between the control and treatment groups. Notably, the outcome increases by about 5.80 when moving from control to treatment, indicating a substantial effect.

**Table 3:** Unadjusted treatment–control mean differences in the simulated mediators and outcome

Variable	Control mean	Treatment mean	Difference
$M_1$	-0.0263	1.0110	1.0373
$M_2$	0.5737	1.3716	0.7979
*Y*	1.0640	6.8667	5.8027

*Key observations:*

- Both mediators are higher in the treatment group, consistent with the direct influence of the randomized intervention.
- The outcome sees a large jump of 5.80, aligning with the total effect estimated in the mediation analysis.

### Sensitivity analyses

We conducted targeted sensitivity analyses aligned with the main sources of fragility in BriDGE: (i) discretization and discovery settings, (ii) outcome-model specification (basis dimension and inclusion of interaction terms), (iii) mediator measurement error, and (iv) violations of mediator–outcome ignorability. Across these checks, the qualitative conclusions in the baseline simulation (direction and magnitude of IDE and JIIE; presence of nonlinearity; and the treatment→mediator ordering implied by randomization) were stable for modest perturbations. Full sensitivity grids, diagnostics, and plots are reported in the Technical Annex (Annex Tables A3–A7 and Annex Fig. A1) and companion materials.

We re-estimated the discovered DAG under a grid of discretization bins and mutual-information test levels to assess how edge orientations change with preprocessing and test thresholds. Results are summarized using overlap metrics (directed-edge Jaccard) and bootstrap arc-strength stability; edges with low stability are reported as ambiguous rather than over-interpreted.

We varied GAM basis dimensions and compared additive-only versus interaction-capable outcome models. When the data-generating process includes mediator synergy and treatment effect modification (Eq. 4), additive-only models can distort the mechanistic decomposition even when TE is estimated reasonably; interaction-capable GAM specifications mitigate this risk.

To reflect behavioral measurement challenges, we perturbed mediator values prior to discovery and mediation estimation. Measurement error reduces discovery stability and can attenuate indirect effects, motivating explicit reporting of mediator reliability (when available) and stress-tests calibrated to plausible reliability ranges.

Although treatment is randomized, mediation identification additionally requires no unmeasured confounding of mediator–outcome relations conditional on measured covariates. Because this assumption is not testable, we report a simulation-based bias analysis that introduces an unobserved confounder with tunable strength and quantifies how IDE/JIIE estimates and coverage change as confounding increases. In behavioral RCTs, this unmeasured confounding can correspond to demand characteristics, common-method bias, or latent motivation affecting both mediator measures and behavioral outcomes; the bias analysis therefore provides a stylized mapping from these threats to their potential impact on IDE/JIIE. The implemented confounder is deliberately additive and should be read as a first-order sensitivity probe rather than as an exhaustive model of behavioral confounding. More complex channels – for example, a latent motivation factor that modifies the mediator–mediator interaction itself – would require application-specific sensitivity models and are discussed as a limitation in the Technical Annex.

This sensitivity analysis is deliberately interpretable rather than exhaustive: the latent factor is modeled as an additive perturbation to mediators and outcome. Behavioral confounding may also operate through nonlinear or interaction channels – for example, a latent motivation factor may alter not only reported mediator values and behavior, but also the synergy between two mediators. We therefore present the additive stress test as a first-order bias probe, and recommend interaction-based sensitivity checks when substantive theory suggests that latent variables may modify mediator–mediator relationships.

Because BCa intervals can be unstable at small *B*, we recommend B≥1000 replicates in typical applications; the simulation uses larger *B* and provides runtime benchmarking templates to support feasibility decisions.

### Semi-synthetic benchmark results (JOBS II scaffold)

We report here the empirical performance of BriDGE on the semi-synthetic benchmark described in Section “Semi-synthetic benchmark on an RCT scaffold (JOBS II)”. We focus on (i) whether constrained discovery recovers the intended mechanistic edges, and (ii) whether the interventional effect decomposition recovers the ground truth under a correctly specified interaction-capable outcome model and how it changes under an additive-only sensitivity specification.

In this benchmark, constrained MMHC recovered the focal mechanistic edges $T\!\rightarrow \!M_1$, $T\!\rightarrow \!M_2$, $T\!\rightarrow \!Y$, $M_1\!\rightarrow \!Y$, and $M_2\!\rightarrow \!Y$. For estimation, we compared a primary interaction-capable outcome GAM against an additive-only sensitivity specification. In line with the semi-synthetic ground truth, the interaction-capable specification closely recovered the interventional direct effect (IDE; truth 2.288, estimate 2.346, 95% BCa [1.986,2.671]), the joint interventional indirect effect (JIIE; truth 3.012, estimate 3.079, 95% BCa [2.657,3.484]), and the total effect (TE; truth 6.360, estimate 6.313, 95% BCa [5.750,6.731]). In contrast, the additive-only specification yielded inflated decomposition components (IDE 2.653; JIIE 3.520) and its IDE interval excluded the true IDE. Consistent with the interventional-effects perspective adopted in BriDGE, we treat mediator-specific marginal indirect effects as secondary in multi-mediator non-additive settings: even under the interaction-capable outcome model, mediator-attribution quantities (e.g., $\textrm{IIE}_{M_1}$ and $\textrm{IIE}_{M_2}$) displayed greater sensitivity than IDE/JIIE/TE, reflecting that, with mediator dependence and outcome interactions, mediator-specific attribution depends on how the joint mediator distribution is perturbed. Accordingly, in applications we recommend emphasizing IDE, TE, and the overall mediated component via JIIE as the primary mechanistic summary, while reporting mediator-specific marginal indirect estimands as descriptive complements rather than definitive “shares” of the total effect.

**Table 4:** Semi-synthetic JOBS II benchmark: Ground truth and BriDGE estimates (95% BCa) for primary interventional effects under the interaction-capable (primary) versus additive-only (sensitivity) outcome GAM

Effect	Truth	Interaction GAM	Additive GAM
IDE	2.288	2.346 [1.986, 2.671]	2.653 [2.404, 2.915]
JIIE	3.012	3.079 [2.657, 3.484]	3.520 [2.921, 3.961]
TE	6.360	6.313 [5.750, 6.731]	6.173 [5.520, 6.614]

Figure 3 shows that, under the randomization constraints, the discovery stage recovers the focal mechanistic edges among treatment, mediators, and outcome. Table 4 confirms that the interaction-capable specification yields small bias and nominal coverage for the direct, total, and overall mediated components, whereas the additive-only specification induces appreciable bias in the decomposition – most clearly for the IDE, whose BCa interval excludes the true value. Full decomposition tables including mediator-specific marginal interventional indirect effects, as well as partial-effect plots and bootstrap histograms for both specifications, are provided in the Online Supplementary Material generated by the companion script.

The key message of this benchmark is that it is possible for the *total* treatment effect to appear similar across specifications while the *mechanistic decomposition* changes meaningfully when non-additive structure is omitted. Here, the interaction-capable GAM recovers the benchmark’s IDE and JIIE within sampling uncertainty, whereas the additive-only model yields an IDE estimate whose BCa interval excludes the true IDE (Table 4). For applied users, this illustrates the practical value of pairing flexible outcome models (GAMs) with specification sensitivity checks: when mechanistic conclusions depend on whether key interactions are included, BriDGE surfaces that dependence explicitly rather than silently imposing additivity.

### Simulation and semi-synthetic benchmark results

Taken together, the simulation study and the semi-synthetic benchmark support two core conclusions about BriDGE. First, when the data-generating structure includes nonlinearities and non-additive features (e.g., treatment–mediator effect modification and mediator–mediator interaction), BriDGE yields a coherent mechanistic decomposition provided that the outcome model is allowed sufficient flexibility. In the simulation setting – where the causal structure and functional relationships are known by design – the protocol produces stable estimates of overall mediated and direct components and offers a transparent way to diagnose discrepancies between the researcher-specified DAG and the empirically discovered graph. In such non-additive settings, it should not be expected that the total effect decomposes exactly into a single direct effect plus a set of mediator-specific path effects; instead, interventional estimands such as the joint interventional indirect effect (JIIE) provide an interpretable summary of the overall mediated component even in the presence of interactions.

Second, the semi-synthetic benchmark clarifies the practical value of BriDGE under realistic data geometry without sacrificing evaluability. By retaining the randomized treatment assignment and baseline covariate distribution of an empirical RCT scaffold (JOBS II) while generating mediators and outcomes from a known structural model, the benchmark simultaneously (i) preserves realistic covariate support and dependence patterns and (ii) maintains a ground truth against which both discovery and mediation estimands can be compared. In this setting, an interaction-capable outcome model closely recovers the interventional direct effect (IDE), the overall mediated component (JIIE), and the total effect (TE), whereas an additive-only outcome specification inflates decomposition components and can yield misleading mechanistic attribution even when the estimated TE remains similar. This contrast illustrates that BriDGE’s modeling flexibility is substantively consequential: it directly shapes conclusions about *how* an intervention operates.

Finally, we emphasize an interpretation principle that applies to both settings. In multi-mediator settings with mediator dependence and outcome interactions, mediator-specific marginal indirect effects are best viewed as secondary descriptive quantities, because their magnitude can depend on how the joint mediator distribution is perturbed. Accordingly, BriDGE places primary emphasis on TE, IDE, and the overall mediated component via JIIE as the most robust mechanistic summaries, complemented by discovery-stage stability diagnostics and sensitivity analyses that probe key modeling and identification assumptions.

**Fig. 4:**
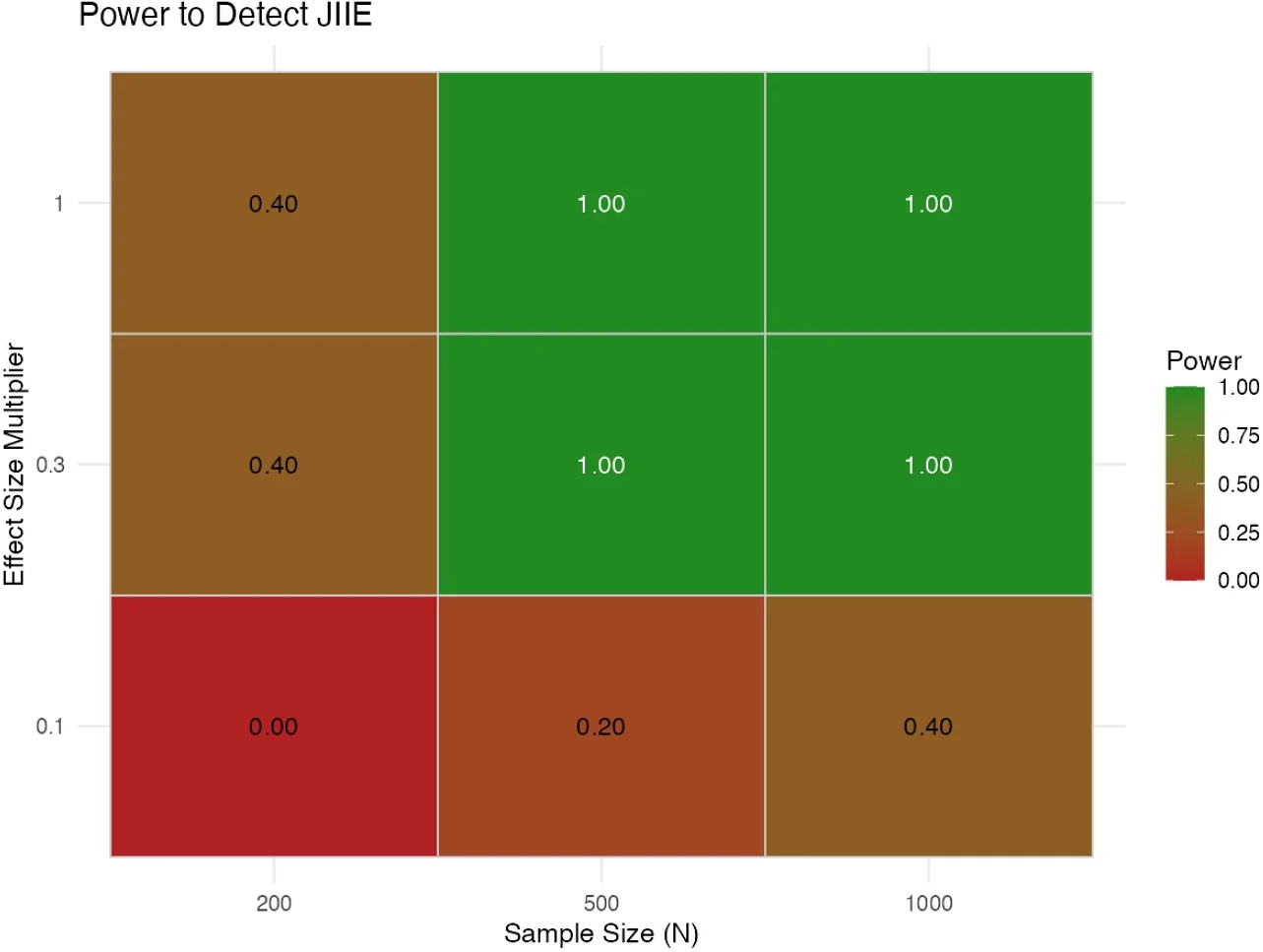
Power to detect the joint interventional indirect effect (JIIE) across sample sizes and effect-size multipliers

## Practical guidance for applied use and feasibility

### Computational feasibility and benchmarking

BriDGE consists of three computationally distinct components: (i) constrained causal discovery on a (possibly discretized) dataset, (ii) estimation of mediator and outcome models (here, GAMs), and (iii) bootstrap-based uncertainty quantification for mediation estimands. In typical applications, the primary computational bottleneck is step (iii), because nonparametric bootstrap inference requires repeated model fitting and repeated Monte Carlo integration.

To make feasibility transparent for prospective users, the companion simulation script benchmarks wall-clock time across sample sizes and mediator dimensionalities for each BriDGE component. We recommend reporting these benchmarks in applied papers to contextualize analytic choices (e.g., choice of *B* and model complexity) and to support reproducibility across hardware.

The bootstrap step dominates computational cost, with runtime growing approximately linearly with *N* and supra-linearly with *p*. Full benchmarking results are provided in the Technical Annex (Annex Table A17 and Annex Fig. A2).

### Sample size and power planning

Both causal discovery stability and mediation inference precision depend strongly on sample size, effect magnitudes, and mediator measurement quality. As a consequence, no single “minimum *N*” is universally appropriate. We therefore recommend simulation-based planning tailored to the hypothesized causal structure and plausible effect sizes, rather than relying solely on generic rules of thumb. The BriDGE codebase includes utilities for power and precision grids that (a) simulate data from user-specified DAGs and functional forms, (b) apply the full BriDGE pipeline, and (c) summarize operating characteristics such as coverage, bias, and the probability that BCa intervals exclude zero for IDE/JIIE.

Figure 4 presents an illustrative power grid as a heatmap, highlighting the interplay between sample size and effect magnitude. The effect-size multiplier simultaneously scales both the treatment-to-mediator ($T\!\rightarrow \!M$) and mediator-to-outcome ($M\!\rightarrow \!Y$) coefficients, so that the indirect effect attenuates quadratically as the multiplier decreases, producing a visible gradient from low to high power. The full numerical power grid is provided in the Technical Annex (Annex Table A18).

### Scaling to multiple mediators and mediator selection

The number of potential mediators in behavioral interventions can be large, and BriDGE must therefore address both statistical and computational scaling. Causal discovery search space grows rapidly with the number of nodes, and conditional independence tests become less stable when conditioning sets are large. Similarly, GAM-based outcome models can become over-parameterized if high-order interactions are included indiscriminately.

In behavioral RCTs, a mediator battery of three to five constructs is often realistic because interventions are hypothesized to operate through multiple psychological and behavioral channels (e.g., beliefs and expectations, intentions, affective responses, perceived norms, and effort or self-regulation). We therefore treat $p\in \{3,5\}$ as substantively motivated intermediate dimensionalities that already create nontrivial discovery and estimation challenges, while remaining typical of mechanism-oriented designs. Moreover, the p=5 mixed scenario explicitly includes *null mediators* to reflect a common empirical pattern: some constructs respond to treatment (and may be informative as manipulation checks or proximal changes) but do not causally transmit effects to the behavioral outcome. In this setting, appropriate performance means distinguishing *treatment responsiveness* (non-zero $T\!\rightarrow \!M$ shifts) from *mechanistic mediation* (near-zero interventional indirect effects).

We recommend a pragmatic, hybrid strategy: (i) *theory-driven screening* to define a defensible candidate mediator set; (ii) *constraint-driven discovery* that encodes temporal ordering (e.g., baseline covariates ≺ mediators ≺ outcomes) via blacklists/whitelists; (iii) *stability selection* (bootstrap arc-strength) to prioritize robust edges; and (iv) *model-complexity management* by limiting interaction smooths to theoretically motivated or stably supported mediator pairs. When *p* is large (e.g., p≥10), additional dimension-reduction or sparse modeling steps (e.g., penalized GAM terms, screening by treatment association, or grouping mediators by construct) may be necessary prior to full BriDGE estimation.

Extended mediation results for p=3 and p=5 (reported in Annex Tables A12–A13 of the Technical Annex) confirm that BriDGE correctly identifies the active indirect pathways (non-zero IIE for true mediators) while assigning near-zero effects to null mediators in the p=5 mixed scenario, demonstrating appropriate mediator-selection behavior as dimensionality grows.

### Comparison with conventional mediation workflows

To clarify the added value of BriDGE relative to commonly used approaches, the simulation script implements a “conventional” mediation baseline based on linear models (single-mediator or parallel-mediator regressions) and product-of-coefficients (or standard causal mediation estimators under linearity). This comparison highlights two recurring issues in behavioral interventions: (i) bias when mediator–outcome relations are nonlinear or when mediator interactions are present, and (ii) mis-calibrated uncertainty when bootstrap distributions are skewed. Substantively, these modeling restrictions can lead applied researchers to misattribute mechanisms – for example, concluding that a single mediator “explains” an effect when the true mechanism depends on thresholding, saturation, or mediator synergy – and thereby misdirect intervention refinement toward components that are not causally decisive (Table 5).

**Table 5:** Simulation comparison (p=2): BriDGE versus conventional linear mediation (Imai’s causal mediation and Sobel test)

Method	Effect	Estimate	CI lo	CI hi
BriDGE	IDE (direct)	2.074	1.923	2.184
BriDGE	JIIE (joint indirect)	3.495	3.305	3.653
BriDGE	TE (total)	7.026	6.736	7.340
BriDGE	IIE (mediator 1)	1.818	1.649	2.050
BriDGE	IIE (mediator 2)	1.050	0.967	1.176
Imai	ACME (mediator 1)	2.474	2.260	2.738
Imai	ACME (mediator 2)	1.781	1.656	1.979
Imai	ADE (direct; averaged)	2.793	Not reported	
Sobel	Indirect (mediator 1)	2.474	2.220	2.727
Sobel	Indirect (mediator 2)	1.781	1.599	1.962

Note. For the conventional Imai model, the ADE is shown as an averaged direct-effect estimate for compact comparison with BriDGE’s IDE. The mediation output returns treatment-regime-specific ADE intervals rather than a single interval for this averaged ADE; therefore, the averaged-ADE confidence interval is not reported

The inflation of the conventional ACME estimates is expected under the interaction-enabled data-generating process. Linear mediation models cannot represent the quadratic mediator effect, the mediator–mediator interaction, or treat-ment–mediator effect modification in Eq. (4). These omitted nonlinear and non-additive components are therefore compressed into average mediator–outcome slopes and then allocated to mediator-specific ACME estimates. Consequently, a larger ACME in the conventional table should not be read as evidence for a stronger single-mediator pathway; rather, it indicates that curvature, mediator synergy, and treatment-effect modification have been conflated with mediator-specific indirect effects. Substantively, this can lead researchers to overstate which psychological construct is driving behavior change and to refine interventions around the wrong mechanism.

For the conventional Imai comparison, direct effects are treatment-regime specific when interaction terms are allowed. Because mediation::mediate returns treatment-regime-specific ADE intervals, a single interval for a compact averaged ADE is not automatically available. Where the comparison table displays an averaged ADE for comparability with BriDGE’s IDE, we therefore state this convention explicitly and treat the averaged-ADE interval as not reported rather than as an unexplained missing confidence interval.

Extended comparisons for the p=3 and p=5 scenarios are provided in the Technical Annex (Annex Tables A15–A16).

For transparency and replicability, we propose a BriDGE reporting checklist (provided in the Technical Annex) covering: the researcher DAG and temporal assumptions; discovery algorithm, constraints, and stability summaries; mediator and outcome model specifications and diagnostics; bootstrap settings and confidence interval type; sensitivity analyses; and clear reporting of IDE/JIIE alongside any mediator-specific marginal indirect estimands.

## Limitations of the BriDGE approach

BriDGE is intended to improve mechanistic understanding by combining (i) explicit causal assumptions via DAGs, (ii) constrained causal discovery for hypothesis interrogation, and (iii) flexible outcome modeling and mediation via GAMs. As with any causal-inference workflow, the validity and interpretability of BriDGE outputs depend on design, measurement quality, and assumptions that cannot be fully verified from the observed data.

First, although randomization guarantees exogeneity of *T*, causal mediation additionally requires assumptions about mediator–outcome relations. Identification of interventional direct and indirect effects typically relies on (i) no unmeasured confounding of each mediator–outcome relationship conditional on measured covariates, together with positivity and consistency, and (ii) an appropriate model for the joint mediator distribution used in g-computation. In behavioral research, mediator–outcome confounding is often plausible even when treatment is randomized (e.g., demand effects, common-method self-report bias, or latent motivation jointly influencing mediator reports and behavior), so BriDGE should be paired with careful baseline covariate measurement (including, where feasible, baseline mediator and outcome measures), pre-specified adjustment sets justified by the DAG, and sensitivity analyses that quantify the robustness of IDE/JIIE to plausible degrees of unmeasured confounding (Technical Annex, Annex Table A5).

The confounding stress-test reported in the Annex (Annex Table A5) uses an additive latent factor because this specification is transparent and easy to calibrate. It should not be interpreted as exhausting the space of behavioral threats to sequential ignorability. In practice, latent demand, motivation, fatigue, social desirability, or common-method processes may also alter nonlinear terms or mediator–mediator interactions; such mechanisms should be represented in application-specific sensitivity analyses when substantive theory suggests them.

Second, causal discovery from finite samples is inherently uncertain. Even under correct assumptions, multiple DAGs can be observationally equivalent (Markov equivalence), and discretization-based conditional-independence testing can induce instability in edge orientation when signal is weak or measurement error is non-negligible. BriDGE therefore treats the discovered DAG as a structured hypothesis generator rather than a definitive mechanistic truth: we recommend enforcing temporal constraints (blacklists/whitelists), reporting bootstrap arc-strength stability, and prioritizing stable structures when refining the researcher DAG. Where discovery and theory conflict, the discrepancy-resolution workflow in Section “Resolving discrepancies between proposed and discovered causal models” provides concrete decision rules.

Third, data requirements and computational costs can be substantial. High-quality mediator measurement, low attrition, and sufficiently large sample sizes are needed for stable discovery and precise mediation inference. Missingness and measurement error are common in behavioral interventions and can attenuate indirect effects and reduce discovery stability; principled missing-data strategies (e.g., multiple imputation or inverse-probability weighting) can be integrated, provided the imputation/weighting model is compatible with the DAG assumptions. Computationally, bootstrap-based inference is typically the dominant cost and scales approximately linearly in the number of bootstrap replicates *B* and nonlinearly with mediator dimensionality, so applied users should balance model complexity against feasibility and report runtime benchmarks.

Finally, extensions may be required in settings with substantial latent confounding, feedback, or temporal dynamics. For example, Partial Ancestral Graphs (PAGs) can represent causal structure under relaxed assumptions and latent variables (Zhang, 2008), albeit with increased interpretational ambiguity. Similarly, longitudinal versions of BriDGE would require explicit modelling of time-varying mediators and outcomes and careful attention to interference and dynamic confounding. These extensions are promising directions for future work.

## Resolving discrepancies between proposed and discovered causal models

Discrepancies between a researcher-specified DAG and a discovered graph are expected in applied work, particularly when mediators are measured with error, temporal separation is imperfect, or conditional-independence tests are sensitive to discretization choices. In BriDGE, such discrepancies are treated as structured evidence for refining (or defending) causal assumptions rather than as a reason to defer to either theory or algorithm by default.

For behavioral researchers, disagreements between theory and discovery are often substantively informative rather than merely technical. They can signal that the theorized mechanism is incomplete, that constructs have been operationalized with error, or that temporal ordering is weaker than assumed (e.g., when mediators and outcomes are measured contemporaneously). We therefore frame stable discrepancies as opportunities for theory refinement: robust edges that persist under temporal constraints and stability checks motivate revisiting the assumed psychological pathway, considering omitted intermediate constructs, and, where feasible, designing follow-up measurements or longitudinal separation that can adjudicate directionality. Conversely, discrepancies that disappear under minor analytic changes or that conflict with design facts should be treated as artifacts rather than as evidence against the underlying theory.

### Recommended workflow

We propose the following iterative procedure:

1. *Enforce temporal and design constraints.* Use blacklists/whitelists to encode what the design makes impossible (e.g., no inbound edges into randomized *T*) and what measurement timing implies (e.g., if *M* precedes *Y*, then blacklist Y→M).
2. *Inspect stability before interpreting orientation.* Summarize discovery uncertainty via bootstrap arc-strength and (when relevant) directionality. Treat edges with low stability as *ambiguous* hypotheses.
3. *Stress-test analytic choices.* Re-run discovery under a small grid of discretization and test-level settings and compare multiple discovery algorithms when feasible (Heinze-Deml et al., 2018; Subramani & Downey, 2017). Discrepancies that vanish under minor analytic changes are unlikely to support strong mechanistic claims. The Technical Annex now includes a weak-signal/noisy-mediator stress-test (Annex Table A7) to demonstrate this step in a setting where algorithms genuinely diverge rather than merely agree on the baseline structure.
4. *Translate robust discrepancies into model refinement.* If a stable discrepancy is theoretically plausible, update the researcher DAG and refit the mediation model; if it is implausible, treat it as a signal of possible assumption violations (e.g., measurement error or unmeasured confounding) and prioritize targeted sensitivity analyses.
5. *Report the full iteration transparently.* Document the original DAG, imposed constraints, discovered graphs, stability summaries, and the rationale for any revisions so that readers can evaluate the inferential path.

The Technical Annex further illustrates this logic with an algorithm-divergence stress test (Annex Table A7). Unlike the baseline simulation, where MMHC, HC, and TABU converge, the stress test weakens the mediator–outcome signal and increases mediator noise to create a genuinely ambiguous orientation problem. The purpose is not to select the algorithm with the most favorable graph, but to show how BriDGE distinguishes stable discrepancies from algorithm-specific artifacts.

### Illustrative example (reversed mediator–outcome edge)

When discovery suggests Y→M but theory implies M→Y, the first diagnostic is measurement timing: if *M* is measured before *Y*, blacklist Y→M and re-run discovery. If the discrepancy persists with correct temporal constraints and is stable under sensitivity to discretization and algorithm choice, then the original theoretical model may be missing an intermediate construct or feedback process. In that case, BriDGE motivates additional measurement, temporal separation, or a longitudinal design to adjudicate directionality.

## Conclusion

Understanding the mechanisms through which behavioral interventions exert their effects is a critical yet challenging endeavor. Our proposed methodological protocol – BriDGE – offers a robust way to enhance mechanistic insights by integrating advanced causal methods (DAGs and GAMs) into randomized controlled trials (RCTs). We believe our stepwise approach to BriDGE provides researchers with a convenient rubric to extend traditional experimental design approaches to uncover and diagnose mechanisms.

By employing DAGs, researchers can make their causal assumptions explicit, facilitating transparent and systematic analysis of the entities and activities that constitute mechanisms. The use of causal discovery algorithms helps uncover the underlying causal structure, providing empirical validation of the hypothesized mechanisms. Generalized additive models (GAMs) enable the modeling of nonlinear relationships and interactions, enhancing the accuracy of effect estimates and capturing complex activities within the mechanism.

BriDGE addresses a significant gap in behavioral science by providing a methodological means to uncover the mechanisms of interventions. It moves beyond the estimation of average treatment effects, offering a nuanced understanding of how interventions work. This mechanistic insight is invaluable for theory development, intervention refinement, and effective implementation in diverse contexts. By providing a structured approach to analyzing complex causal pathways, our protocol complements recent proposals for more integrative approaches to experimental design in the social and behavioral sciences (Almaatouq et al., 2024).

By enhancing the transparency and rigor of causal analysis, BriDGE contributes to the advancement of behavioral science research. It empowers researchers to make more informed decisions about which components of an intervention are most effective, how the activities produce change, and how to adapt interventions for different populations, thereby building more robust behavioral theories.

The application of BriDGE to fully empirical RCT data will be the next critical step. Such studies cannot validate graph recovery in the same way as simulations or semi-synthetic benchmarks, because the true causal graph is unknown; however, they can demonstrate feasibility, interpretability, and practical implementation under naturally occurring missingness, measurement error, and construct-validity constraints. Further methodological refinement may also incorporate other advanced causal inference techniques, such as machine learning algorithms for causal discovery or methods for handling high-dimensional data.

Finally, for practitioners and policymakers, the enhanced mechanistic understanding provided by BriDGE can inform more targeted and effective interventions. By knowing not just that an intervention works, but *how and why* it works, practitioners can adapt strategies to local contexts, optimize resource allocation, and improve outcomes, harnessing the pluralism that behavioral science has to offer (Banerjee & Veltri, 2024).

## Supplementary Information

Below is the link to the electronic supplementary material.

Supplementary file 1 (pdf 687 KB)

## Data Availability

The simulation data are generated by the companion code; the semi-synthetic benchmark uses a publicly available JOBS II scaffold. Documentation about the code is available at this link OSF anonymized repository.

## Declarations

### Software availability

All analyses were conducted in R. We provide bridgeR, an open-source package that implements the BriDGE workflow used here (data generation, constrained MMHC discovery, GAM estimation, and mediation with bootstrap inference). The code used for the simulation and all figures and tables is included as package vignettes. The R code is available at this link OSF anonymized repository.
