# Supplementary material for: BriDGE the gap: Improving behavioral research by integrating DAGs and GAMs into experiments
Source: Behav Res Methods. 2026 Sep 23;58(11):298. doi: 10.3758/s13428-026-03146-2 (PMC13601132; doi:10.3758/s13428-026-03146-2)
Supplement: Supplementary file 1 [file 13428_2026_3146_MOESM1_ESM.pdf]

Technical Annex

Companion to: BriDGE – Bridging Discovery, GAMs, and Estimation for Causal Mediation in RCTs

1 Introduction

Randomized Controlled Trials (RCTs) are considered the gold standard in establishing causal relationships due to their ability to minimize confounding through random assignment of treatment. However, even in RCTs, understanding the complex mechanisms underlying treatment effects, particularly when nonlinear relationships and interactions are present, remains a challenge. Traditional linear models may not adequately capture these complexities, potentially leading to biased or incomplete conclusions.

This analysis presents a comprehensive methodology that integrates nonlinear modeling techniques with causal inference methods to enhance the validity of findings in RCTs. By leveraging Generalized Additive Models (GAMs) and causal discovery algorithms, we aim to uncover intricate causal structures that may be overlooked by conventional approaches. Furthermore, combining causal discovery with traditional causal inference provides a robust framework for validating hypothesized causal relationships and identifying potential discrepancies.

The primary objectives of this analysis are to:

1. **Data Generation**: Generate synthetic data based on a researcher's proposed causal Directed Acyclic Graph (DAG), incorporating both linear and nonlinear relationships to mimic real-world complexities.
2. **Causal Discovery**: Apply causal discovery algorithms to evaluate how well the discovered DAG aligns with the researcher's DAG, thereby assessing the validity of the initial causal assumptions.
3. **Causal Mediation Analysis**: Perform causal mediation analysis using GAMs to quantify direct and indirect effects of the treatment on the outcome through mediators, capturing nonlinear effects.
4. **Sensitivity Analysis**: Assess the robustness of the findings through sensitivity analysis, evaluating the impact of small perturbations in the mediators on the estimated effects.

By addressing nonlinear relationships and integrating multiple causal analysis techniques, this methodology aims to provide more accurate and reliable insights into the causal mechanisms within RCTs, ultimately enhancing the interpretation and generalizability of the results.

2 Data Generation

2.1 Variables

The primary variables in the analysis are:

- **Treatment (T)**: A binary variable indicating assignment to the treatment ($T = 1$) or control group ($T = 0$).
- **Mediators (M_1, M_2)**: Variables that may mediate the effect of the treatment on the outcome.
- **Outcome (Y)**: The primary outcome variable of interest.

2.2 Data Generation Process

We generate synthetic data reflecting both linear and nonlinear relationships, as well as interactions, to mimic complexities found in real-world data.

1. **Treatment Assignment**:

$$T \sim \text{Bernoulli}(0.5) \tag{1}$$

2. **Mediator Variables**:

$$M_1 = \beta_{TM_1} T + \epsilon_{M_1} + \text{NL}_1 \tag{2}$$
$$M_2 = \beta_{TM_2} T + \epsilon_{M_2} + \text{NL}_2 \tag{3}$$

where:

- β_{TM_1} and β_{TM_2} are coefficients for the effect of T on M_1 and M_2, respectively.
- $\epsilon_{M_1}, \epsilon_{M_2} \sim \mathcal{N}(0, 1)$ are noise terms.
- $\text{NL}_1 = \text{nonlinear\_strength} \times \sin(Z_1)$, with $Z_1 \sim \mathcal{N}(0, 1)$.
- $\text{NL}_2 = \text{nonlinear\_strength} \times \exp(Z_2/2)$, with $Z_2 \sim \mathcal{N}(0, 1)$.

Rationale for Nonlinear Functions:

The inclusion of nonlinear components NL_1 and NL_2 in the mediator variables serves to simulate complex, real-world relationships that are not adequately captured by linear models alone. The specific choices of the sine function for NL_1 and the exponential function for NL_2 are motivated by the following considerations:

- **Sine Function in NL_1**:
  - The sine function introduces periodicity and oscillatory behavior into M_1.
  - This reflects real-world phenomena where variables exhibit cyclical patterns, such as seasonal effects, biological rhythms, or economic cycles.
  - Incorporating the sine function allows us to model mediator effects that vary in a nonlinear, non-monotonic fashion relative to underlying factors.
- **Exponential Function in NL_2**:
  - The exponential function introduces rapid growth or decay into M_2, capturing relationships where changes in an underlying factor lead to exponential increases or decreases.
  - This reflects scenarios such as population growth, spread of diseases, or financial interest accumulation.
  - Including the exponential function allows us to simulate mediator effects that are sensitive to small changes in underlying factors, resulting in large variations in M_2.

By integrating these nonlinear functions, we create mediators that exhibit complex behaviors commonly observed in various fields, including economics, biology, and social sciences. This enhances the realism of the synthetic data and challenges analytical methods to accurately capture and model such complexities.

3. **Outcome Variable**:

$$\begin{aligned} Y = {} & \beta_{TY} T + \beta_{M_1 Y} M_1 + \beta_{M_2 Y} M_2 + \gamma_{M_1^2} M_1^2 + \gamma_{M_1 M_2} M_1 M_2 \\ & + \gamma_{TM_1} T M_1 + \gamma_{TM_2} T M_2 + \epsilon_Y \end{aligned} \tag{4}$$

where:

- $\beta_{TY}, \beta_{M_1Y}, \beta_{M_2Y}$ are coefficients for direct effects.
- $\gamma_{M_1^2}, \gamma_{M_1M_2}, \gamma_{TM_1}, \gamma_{TM_2}$ are coefficients for higher-order and interaction effects.
- $\epsilon_Y \sim \mathcal{N}(0,1)$ is a noise term.

Rationale for Outcome Model Specification:

The outcome variable Y is modelled as a function of both linear and nonlinear terms, including interactions and higher-order effects. This reflects the reality that outcomes in many fields are influenced by complex interplays between variables, not just by simple additive effects. The inclusion of terms like M_1^2 and M_1M_2 allows us to capture:

- **Nonlinear Effects of Mediators**:
  - Squared terms represent situations where the effect of a mediator increases or decreases at an accelerating rate.
  - This can model phenomena such as diminishing returns or threshold effects.
- **Interactions Between Mediators**:
  - Interaction terms like M_1M_2 capture the combined effect of mediators acting together on the outcome.
  - This reflects scenarios where the impact of one mediator depends on the level of another, a common occurrence in systems with synergistic or antagonistic relationships.
- **Interactions with Treatment**:
  - Terms like TM_1 and TM_2 allow the effect of the treatment to vary depending on the level of the mediators.
  - This models effect modification, where the treatment effect is not uniform across all individuals but depends on other factors.

By incorporating these complexities into the outcome model, we create a challenging environment for causal analysis methods, ensuring that the methodology can handle realistic data structures with nonlinearities and interactions.

2.3 Directed Acyclic Graphs (DAGs)

Directed Acyclic Graphs (DAGs) are graphical representations of causal relationships between variables (Pearl, 1995). In a DAG, nodes represent variables, and directed edges (arrows) indicate causal influences from one variable to another. The "acyclic" nature means that the graph does not contain any feedback loops; you cannot start at one node and follow a directed path that leads back to the starting node.

Let $\mathbf{X} = \{X_1, X_2, \ldots, X_p\}$ represent a set of random variables. A DAG $\mathcal{G}$ is a directed graph with nodes corresponding to the variables $\mathbf{X}$, and directed edges representing causal relationships between them. The structure of a DAG encodes conditional independencies among variables using the following key rule: A variable X_i is conditionally independent of its non-descendants given its parents in the graph. This is known as the Markov Property for DAGs (Pearl, 2009).

For instance, consider the following simple DAG, which models the relationships between a treatment, a mediator, and an outcome:

$$\text{Treatment} \rightarrow \text{Mediator} \rightarrow \text{Outcome}$$

In this case, the treatment variable directly influences the mediator, and the mediator, in turn, influences the outcome. The absence of an edge from the treatment directly to the outcome indicates that the treatment affects the outcome *only* through the mediator.

DAGs allow us to formalize causal assumptions and derive the implied conditional independencies. The d-separation criterion is a graphical criterion used to determine whether two sets of nodes are independent given a third set. Formally, given a DAG $\mathcal{G}$, two sets of variables A and B are conditionally independent given a third set C (denoted $A \perp B \mid C$) if C d-separates A from B in the graph (Pearl, 1995). D-separation can be used to identify confounders, mediators, and colliders in a causal model.

DAGs can also be represented through a system of structural equations, known as a Structural Equation Model (SEM). In SEM, each variable X_i is expressed as a deterministic function of its parent variables $\text{PA}(X_i)$ and a stochastic noise term ϵ_i:

$$X_i = f_i(\text{PA}(X_i), \epsilon_i),$$

where ϵ_i is an independent error term. This system of equations defines the causal mechanisms governing the relationships between variables.

The primary use of DAGs in causal inference is to identify and adjust for confounders. Given a DAG $\mathcal{G}$, we can determine whether a set of covariates $\mathbf{Z}$ is sufficient to adjust for confounding when estimating the causal effect of a treatment T on an outcome Y. The backdoor criterion provides a formal rule for identifying such covariates (Pearl, 2009):

- A set of covariates $\mathbf{Z}$ satisfies the backdoor criterion relative to (T, Y) if $\mathbf{Z}$ blocks every path between T and Y that contains an arrow into T (i.e., a backdoor path).

In summary, DAGs provide a graphical framework for representing causal assumptions and guiding the identification of confounders, mediators, and colliders. By encoding these relationships, DAGs allow researchers to make informed decisions about which variables to adjust for, and which pathways to explore, in order to estimate causal effects accurately. DAGs serve as the visual and theoretical backbone of the causal inference process, guiding subsequent analyses such as constrained causal discovery, GAM-based outcome modelling, and interventional mediation analysis.

3 Causal Discovery Using Nonlinear Methods

3.1 Algorithm Selection

We use the **Max-Min Hill Climbing (MMHC)** algorithm from the `bnlearn` package, a hybrid method combining constraint-based and score-based approaches, suitable for learning Bayesian networks. We additionally triangulate discovery results using Hill Climbing (HC) and TABU search algorithms under the same constraint structure to assess algorithmic robustness (see Section 6.6).

3.2 Implementation Steps

1. **Data Preprocessing**:
   - Ensure variables are correctly typed (e.g., factors for categorical variables, numerics for continuous variables).
   - Discretize continuous variables using equal-frequency quantiles (default $g = 5$ bins) for mutual-information testing in constraint-based phases.
2. **Structure Learning**:
   - Apply the MMHC algorithm to the processed data to learn the DAG structure.
   - Incorporate RCT design knowledge via blacklists (no inbound edges to treatment) and whitelists (treatment $\rightarrow$ mediator edges).
3. **DAG Creation**:
   - Extract the adjacency matrix from the MMHC result.
   - Create the discovered DAG using the adjacency matrix.
4. **DAG Comparison**:
   - Compare the edges in the discovered DAG with those in the researcher's proposed DAG.
   - Identify agreements and discrepancies.
   - Compute SHD, precision, recall, F1 score, and bootstrap arc-strength stability.

4 DAG Comparison

4.1 Objective

To assess how well the causal structure learned from the data (the **discovered DAG**) aligns with the causal structure hypothesized by the researcher (the **researcher's DAG**). This comparison helps validate the researcher's assumptions and identifies any discrepancies that may require further investigation.

4.2 Methodology

The comparison involves several steps to quantitatively and qualitatively evaluate the similarities and differences between the two DAGs.

1. **Representation of DAGs**:
   - The **researcher's DAG** (G_R) is constructed based on the hypothesized causal relationships. It includes nodes representing variables and directed edges indicating assumed causal effects.
   - The **discovered DAG** (G_D) is obtained from the causal discovery algorithm applied to the data.
2. **Extraction of Edge Lists**:
   - Extract the set of directed edges (arcs) from both G_R and G_D.
   - Represent each edge as an ordered pair ($X_i \to X_j$), where X_i is the parent (cause) and X_j is the child (effect).
   - Denote the edge sets as:

$$E_R = \text{Edge set of } G_R$$
$$E_D = \text{Edge set of } G_D$$

3. **Creation of Comparison Data Frame**:
   - Combine the edge lists into a unified data frame or table that records the presence or absence of each possible edge in both DAGs.
   - For each possible edge between variables, create indicators:

$$\text{Researcher Indicator (RI)} = \begin{cases} 1, & \text{if edge is in } E_R \\ 0, & \text{otherwise} \end{cases}$$

$$\text{Discovered Indicator (DI)} = \begin{cases} 1, & \text{if edge is in } E_D \\ 0, & \text{otherwise} \end{cases}$$

4. **Identification of Agreements and Discrepancies**:
   - **True Positives (TP)**: Edges present in both G_R and G_D (RI = 1 and DI = 1).
   - **False Negatives (FN)**: Edges present in G_R but absent in G_D (RI = 1 and DI = 0).
   - **False Positives (FP)**: Edges absent in G_R but present in G_D (RI = 0 and DI = 1).
   - **True Negatives (TN)**: Edges absent in both G_R and G_D (RI = 0 and DI = 0).
5. **Quantitative Metrics**:
   - **Edge Agreement Rate**:

$$\text{Agreement Rate} = \frac{\text{Number of TPs}}{\text{Number of Edges in } G_R} \times 100\%$$

- **Precision**:
$$\text{Precision} = \frac{\text{Number of TPs}}{\text{Number of TPs} + \text{Number of FPs}}$$
- **Recall**:
$$\text{Recall} = \frac{\text{Number of TPs}}{\text{Number of TPs} + \text{Number of FNs}}$$
- **F1 Score**:
$$\text{F1 Score} = 2 \times \frac{\text{Precision} \times \text{Recall}}{\text{Precision} + \text{Recall}}$$
- These metrics provide a quantitative assessment of how well the discovered DAG matches the researcher's DAG.

6. **Visualization of Differences**:
   - Plot both DAGs side by side using graphical tools.
   - Use color-coding or edge styles to highlight:
     - Edges common to both DAGs (e.g., solid lines).
     - Edges only in the researcher's DAG (e.g., dashed red lines).
     - Edges only in the discovered DAG (e.g., dotted lines).
7. **Interpretation of Results**:
   - Analyze the agreements to confirm the validity of the researcher's assumptions.
   - Investigate discrepancies to understand potential reasons, such as:
     - Statistical insignificance of certain relationships.
     - Overfitting or underfitting in the causal discovery algorithm.
     - Presence of confounding variables not accounted for.
     - Model misspecification in the researcher's DAG.
   - Consider whether discrepancies warrant revising the researcher's causal model or further data collection.

4.3 Implementation Details

In practice, the DAG comparison is implemented using the following steps in the code:

1. **Edge Extraction**:
   - For each DAG, extract the edge list using functions like `as_edgelist()` from the `igraph` package.
   - Convert the edge lists into data frames with columns `From` and `To`.
2. **Creation of Indicator Variables**:
   - Add a column to each data frame indicating the source of the edge (`Researcher` or `Discovered`).
   - For the researcher's DAG edges, set `Researcher` = TRUE.
   - For the discovered DAG edges, set `Discovered` = TRUE.
3. **Merging Edge Data Frames**:
   - Perform a full outer join on the `From` and `To` columns to combine the two edge data frames.
   - Replace missing values with FALSE in the indicator columns.
4. **Classification of Edges**:
   - Add a column `EdgeType` to classify each edge based on the indicator variables:

- "TP" if `Researcher` = TRUE and `Discovered` = TRUE.
- "FN" if `Researcher` = TRUE and `Discovered` = FALSE.
- "FP" if `Researcher` = FALSE and `Discovered` = TRUE.
- "TN" if `Researcher` = FALSE and `Discovered` = FALSE (typically not included in the edge list as no edge exists).

5. **Calculation of Metrics**:
   - Count the number of edges in each category (TP, FN, FP).
   - Compute precision, recall, F1 score, and agreement rate as per the formulas provided.
6. **Visualization**:
   - Use plotting functions to visualize the combined DAG, highlighting edges based on their classification.
   - Include legends and annotations to explain the color-coding or edge styles.
7. **Reporting**:
   - Present the metrics in a table or summary format.
   - Discuss the implications of the findings in the context of the research question.

4.4 Example

Suppose we have the following:

- **Researcher's DAG Edges** (E_R):

$$\begin{aligned} T &\to M_1 \\ T &\to M_2 \\ M_1 &\to Y \\ M_2 &\to Y \\ T &\to Y \end{aligned}$$

- **Discovered DAG Edges** (E_D):

$$\begin{aligned} T &\to M_1 \\ M_1 &\to Y \\ M_2 &\to Y \\ M_1 &\to M_2 \end{aligned}$$

Edge Classification:

From	To	Edge Type
T	M_1	TP
T	M_2	FN
M_1	Y	TP
M_2	Y	TP
T	Y	FN
M_1	M_2	FP

Annex Table A1: Classification of Edges

Metrics Calculation:

- Number of TPs = 3
- Number of FNs = 2
- Number of FPs = 1
- Precision:

$$\text{Precision} = \frac{3}{3+1} = \frac{3}{4} = 0.75$$

- Recall:

$$\text{Recall} = \frac{3}{3+2} = \frac{3}{5} = 0.6$$

- F1 Score:

$$\text{F1 Score} = 2 \times \frac{0.75 \times 0.6}{0.75 + 0.6} \approx 0.6667$$

- Agreement Rate:

$$\text{Agreement Rate} = \frac{3}{5} \times 100\% = 60\%$$

4.5 Interpretation

The discovered DAG correctly identified 60% of the edges proposed by the researcher. The presence of false negatives indicates that some relationships hypothesized by the researcher were not detected by the algorithm. The false positive edge ($M_1 \to M_2$) suggests that the algorithm identified a potential causal relationship not considered by the researcher.

Possible explanations for discrepancies include:

- **False Negatives**:
  - The effects of T on M_2 and Y may be weak or masked by noise.
  - Sample size may be insufficient to detect certain relationships.
  - Algorithm limitations in capturing complex interactions or nonlinearities.
- **False Positives**:
  - The algorithm may have detected a spurious relationship due to random variation.
  - There may be an unconsidered causal pathway from M_1 to M_2.

4.6 Limitations

- **Causal Discovery Algorithm Constraints**:
  - Algorithms like MMHC may not capture all types of causal relationships, especially in the presence of nonlinearities or latent variables.
- **Equivalence Classes**:
  - Different DAGs can be Markov equivalent, meaning they encode the same conditional independence relationships and cannot be distinguished based on observational data alone.
- **Sample Size and Noise**:
  - Limited data or high noise levels can reduce the power to detect true causal relationships.
- **Assumptions Violations**:
  - Violations of causal discovery algorithm assumptions (e.g., causal sufficiency, faithfulness) can lead to incorrect inferences.

4.7 Conclusion

The DAG comparison provides a structured approach to evaluating the alignment between the researcher's hypothesized causal model and the structure learned from the data. By quantifying agreements and discrepancies, researchers can:

- Validate or question their initial causal assumptions.
- Identify areas where the model may need refinement.
- Gain insights into the data that may inform further analysis or experimentation.

This step is crucial in causal analysis, especially in the context of RCTs, as it bridges theoretical assumptions with empirical evidence.

4.8 Quantitative Metrics

In the current BRIDGE implementation, DAG comparison employs multiple quantitative metrics:

- **Structural Hamming Distance (SHD)**: The number of edge additions, deletions, and reversals needed to transform the discovered DAG into the researcher DAG. SHD = 0 indicates perfect structural recovery.
- **Precision**: $\text{Precision} = \text{TP}/(\text{TP} + \text{FP})$
- **Recall**: $\text{Recall} = \text{TP}/(\text{TP} + \text{FN})$
- **F1 Score**: $\text{F1} = 2 \times \text{Precision} \times \text{Recall}/(\text{Precision} + \text{Recall})$
- **Bootstrap Arc-Strength Stability**: For each discovered edge, the proportion of bootstrap resamples in which that edge appears, providing a measure of discovery uncertainty. Edges with low arc-strength are treated as ambiguous.

These metrics are computed automatically by the BRIDGE simulation script for each scenario ($p \in \{2, 3, 5\}$) and reported alongside directed-edge overlap tables.

4.9 Edge Classification

The R code identifies edges where there is disagreement between the DAGs by comparing the `Researcher` and `Discovered` indicators. Edges where these indicators differ highlight discrepancies that warrant further investigation. In the revised implementation, discrepancies are further classified using the concrete resolution workflow described in the main manuscript (Section 4.10).

4.10 Discrepancy Resolution Workflow

When the discovered graph differs from the researcher DAG, we recommend the following iterative workflow:

1. **Classify the discrepancy**: missing edges, extra edges, or orientation conflicts.
2. **Enforce design and temporal constraints**: blacklists/whitelists encoding what is logically impossible.
3. **Quantify discovery uncertainty**: bootstrap arc-strength summaries.
4. **Stress-test analytic choices**: re-run discovery under plausible discretization and test-level settings (Annex Table A3).
5. **Triangulate across algorithms**: compare MMHC, HC, TABU under the same constraints (Annex Table A6).
6. **Translate discrepancies into model refinement or sensitivity analyses**.
7. **Document the iteration transparently**.

5 Causal Mediation Analysis Using GAMs

5.1 Objective

To decompose the total effect of the treatment on the outcome into direct and indirect effects mediated by $M_1, \ldots, M_p$, while capturing nonlinear relationships using Generalized Additive Models (GAMs). The BriDGE framework uses *interventional* mediation estimands—the Interventional Direct Effect (IDE), the Joint Interventional Indirect Effect (JIIE), and per-mediator Interventional Indirect Effects (IIE)—which remain well-defined in the presence of multiple mediators and treatment–mediator interactions, unlike classical natural direct/indirect effects that require the cross-world independence assumption.

5.2 Estimation Procedure

Mediation effects are estimated via Monte-Carlo g-computation using the fitted mediator and outcome models. The procedure involves fitting separate models for each mediator and the outcome, then computing expected outcomes under counterfactual mediator distributions.

5.2.1 Mediator Models

Because T is binary and randomized, we model each mediator with a parsimonious linear regression on T:

$$M_j = \beta_{0,M_j} + \beta_{T,M_j}\,T + \epsilon_{M_j}, \qquad j \in \{1, \ldots, p\} \tag{5}$$

where $\epsilon_{M_j} \sim \mathcal{N}(0, \sigma^2_{M_j})$ are noise terms.

5.2.2 Outcome Model

In the baseline two-mediator simulation ($p = 2$), the data-generating process includes nonlinear main effects, mediator–mediator interaction, and treatment–mediator effect modification. To capture these features we fit the following GAM:

$$Y = \beta_{0,Y} + \beta_{T,Y}\,T + s_1(M_1) + s_2(M_2) + ti(M_1, M_2) + s_1(M_1,\, \text{by} = T) + s_2(M_2,\, \text{by} = T) + \varepsilon_Y \tag{6}$$

where:

- $s_j(\cdot)$ are thin-plate regression splines estimated using `mgcv`.
- $ti(\cdot, \cdot)$ denotes a tensor-product interaction smooth.
- The "by" smooths allow treatment-specific deviations in the mediator effects (effect modification).
- ε_Y is the error term.

Implementation details: GAMs are fit in R using *mgcv* Wood (2011, 2017) with Gaussian family (identity link), thin-plate regression splines, smoothing parameters estimated via REML, and default basis dimensions (e.g., $k = 10$) subject to diagnostic checks (e.g., `gam.check`).

5.2.3 Estimation of Expected Outcomes

Compute the expected outcomes under different scenarios:

1. **Estimate Mediator Distributions:**
   - Use the mediator models to predict $M_{j,T=0}$ and $M_{j,T=1}$, the expected values of M_j when $T = 0$ and $T = 1$, respectively, for each mediator j.
2. **Compute Expected Outcomes via Monte-Carlo Integration:**

- For each required expectation, input the appropriate values of T and $\{M_1, \ldots, M_p\}$ into the outcome model to obtain $\mathbb{E}[Y]$.
- Monte-Carlo integration is used to average over the empirical mediator distributions, drawing mediator values from the fitted mediator models under the appropriate treatment condition.

5.3 Effect Estimation: Interventional Estimands

Calculate the mediation effects using interventional estimands:

1. **Interventional Direct Effect (IDE)**:

$$\text{IDE} = \mathbb{E}[Y_{T=1,\mathbf{M}=\mathbf{M}_{T=0}}] - \mathbb{E}[Y_{T=0,\mathbf{M}=\mathbf{M}_{T=0}}] \tag{7}$$

   where $\mathbf{M}_{T=0}$ denotes the joint mediator distribution under control. The IDE captures the effect of treatment holding all mediators at their control-condition distributions.

2. **Joint Interventional Indirect Effect (JIIE)**:

$$\text{JIIE} = \mathbb{E}[Y_{T=0,\mathbf{M}=\mathbf{M}_{T=1}}] - \mathbb{E}[Y_{T=0,\mathbf{M}=\mathbf{M}_{T=0}}] \tag{8}$$

   The JIIE captures the joint effect of shifting all mediator distributions from their control to their treatment distributions, while holding treatment itself at control. This is the *primary* indirect summary.

3. **Per-mediator Interventional Indirect Effect ($\textbf{IIE}_{M_j}$)**:

$$\text{IIE}_{M_j} = \mathbb{E}[Y_{T=0,M_j=M_{j,T=1},M_{-j}=M_{-j,T=0}}] - \mathbb{E}[Y_{T=0,\mathbf{M}=\mathbf{M}_{T=0}}] \tag{9}$$

   The IIE_{M_j} isolates the indirect effect through mediator j by shifting only M_j to its treatment distribution while holding all other mediators at their control distributions. **Note:** because mediator–mediator interactions imply that marginal indirect effects need not sum to the JIIE, the per-mediator IIEs are treated as complementary diagnostics rather than an additive decomposition.

4. **Total Effect (TE)**:

$$\text{TE} = \mathbb{E}[Y_{T=1,\mathbf{M}=\mathbf{M}_{T=1}}] - \mathbb{E}[Y_{T=0,\mathbf{M}=\mathbf{M}_{T=0}}] \tag{10}$$

5.4 Bootstrap Inference: BCa Confidence Intervals

To estimate the variability of the effects and construct confidence intervals, we employ nonparametric bootstrapping with *bias-corrected and accelerated* (BCa) intervals rather than standard percentile intervals. BCa intervals are preferred because mediation estimands frequently yield asymmetric and biased bootstrap sampling distributions, especially under nonlinear outcome models.

5.4.1 BCa Interval Construction

The BCa method adjusts the percentile bootstrap for two sources of error:

1. **Bias correction ($\hat{z}_0$):** The bias-correction factor measures the median bias of the bootstrap distribution relative to the point estimate. It is estimated as:

$$\hat{z}_0 = \Phi^{-1}\left(\frac{1}{B}\sum_{b=1}^{B} \mathbf{1}\left[\hat{\theta}^{*b} < \hat{\theta}\right]\right) \tag{11}$$

   where $\hat{\theta}$ is the point estimate, $\hat{\theta}^{*b}$ are bootstrap replicates, and Φ^{-1} is the standard normal quantile function.

2. **Acceleration ($\hat{a}$):** The acceleration factor accounts for the rate at which the standard error of $\hat{\theta}$ changes with θ. It is estimated using a delete-group jackknife:

$$\hat{a} = \frac{\sum_{g=1}^{G} \left(\hat{\theta}_{(\cdot)} - \hat{\theta}_{(-g)}\right)^3}{6\left[\sum_{g=1}^{G} \left(\hat{\theta}_{(\cdot)} - \hat{\theta}_{(-g)}\right)^2\right]^{3/2}} \tag{12}$$

where $\hat{\theta}_{(-g)}$ is the estimate with the g-th group deleted and $\hat{\theta}_{(\cdot)}$ is the mean of all jackknife estimates. For efficiency, the jackknife omits GAM refitting and instead re-averages predictions from the full-sample GAMs on the leave-group-out subsets, using $G = 50$ groups.

3. **Adjusted percentiles:** The $(1-\alpha)$ BCa interval uses adjusted quantiles:

$$\alpha_1 = \Phi\left(\hat{z}_0 + \frac{\hat{z}_0 + z_{\alpha/2}}{1 - \hat{a}(\hat{z}_0 + z_{\alpha/2})}\right), \quad \alpha_2 = \Phi\left(\hat{z}_0 + \frac{\hat{z}_0 + z_{1-\alpha/2}}{1 - \hat{a}(\hat{z}_0 + z_{1-\alpha/2})}\right) \tag{13}$$

and the interval is $\left[\hat{\theta}^{*(\alpha_1)}, \hat{\theta}^{*(\alpha_2)}\right]$.

5.4.2 Recommended Bootstrap Settings

For numerical stability of BCa intervals, we recommend at least $B \geq 1000$ bootstrap replicates (we use $B = 2000$ in the simulation). Annex Table A2 compares interval widths and stability across different values of B.

Annex Table A2: Bootstrap replicate sensitivity: BCa interval width and stability as a function of B.

B	IDE_width	JIIE_width	JIIE_lo	JIIE_hi	Runtime_s	n_failed
20.000	0.191	0.468	3.352	3.820	3.200	0.000
50.000	0.303	0.511	3.337	3.848	11.600	0.000

5.5 Assumptions

The validity of the mediation analysis relies on several key assumptions:

- **No Unmeasured Confounding (Sequential Ignorability):**
  - There are no unmeasured confounders affecting the relationships between T and M_j, or between M_j and Y, conditional on measured covariates.
  - Although treatment is randomized, causal mediation additionally requires that there are no unmeasured confounders of each mediator–outcome relation conditional on measured covariates. Because this assumption is not testable, we provide a simulation-based bias analysis (Section 6.5).
- **Correct Model Specification:**
  - The GAMs correctly specify the functional form of the relationships between variables, capturing all relevant nonlinearities and interactions.
  - GAM specification sensitivity is assessed by varying the basis dimension (e.g., $k \in \{6, 10, 20\}$) and comparing model diagnostics via `gam.check()`.
- **Positivity:**
  - There is sufficient overlap in the distributions of the mediators and treatment levels, allowing for valid estimation across different combinations of T and $\{M_1, \ldots, M_p\}$.
- **Consistency:**
  - The observed outcomes correspond to the potential outcomes under the observed treatment and mediator values.

5.6 Interpretation of Results

By estimating the IDE, JIIE, per-mediator IIEs, and TE, we can quantify:

- The direct effect of the treatment on the outcome not mediated through the measured mediators (IDE).
- The joint indirect effect through all mediators simultaneously (JIIE), which is the primary indirect summary.
- The marginal indirect contribution of each individual mediator (IIE_{M_j}), with the caveat that these need not sum to the JIIE when mediator interactions are present.
- The proportion of the total effect explained by the mediators.

Understanding these effects provides insights into the mechanisms by which the treatment influences the outcome and the relative importance of each mediator.

6 Sensitivity Analysis

6.1 Objective

To assess the robustness of the mediation results across the main sources of fragility in BriDGE: (i) discretization and discovery settings, (ii) GAM specification, (iii) mediator measurement error, and (iv) potential mediator–outcome confounding (violations of sequential ignorability).

6.2 Discovery Sensitivity: Discretization and Test-Level Choices

The MMHC causal discovery algorithm requires discretization of continuous variables for mutual-information testing. Since the choice of number of bins g and test significance level α are somewhat arbitrary, we assess sensitivity by re-running MMHC over a grid of $g \in \{3, 4, 5, 6, 7\}$ bins and computing (a) structural Hamming distance (SHD) to the researcher DAG, (b) directed-edge precision, recall, and F1 score, and (c) mediation estimand stability. A two-panel visualisation (Annex Figure A1) shows both DAG recovery metrics and effect estimate stability across the discretization grid.

Annex Table A3: Discretization sensitivity summary.

bins	SHD	precision	recall	f1	n_arcs	IDE	JIIE	TE
3.000	4.000	0.833	1.000	0.909	7.000	2.074	3.495	7.026
4.000	4.000	0.833	1.000	0.909	7.000	2.074	3.495	7.026
5.000	4.000	0.833	1.000	0.909	7.000	2.074	3.495	7.026
6.000	4.000	0.833	1.000	0.909	7.000	2.074	3.495	7.026
7.000	0.000	0.800	0.800	0.800	5.000	2.074	3.495	7.026

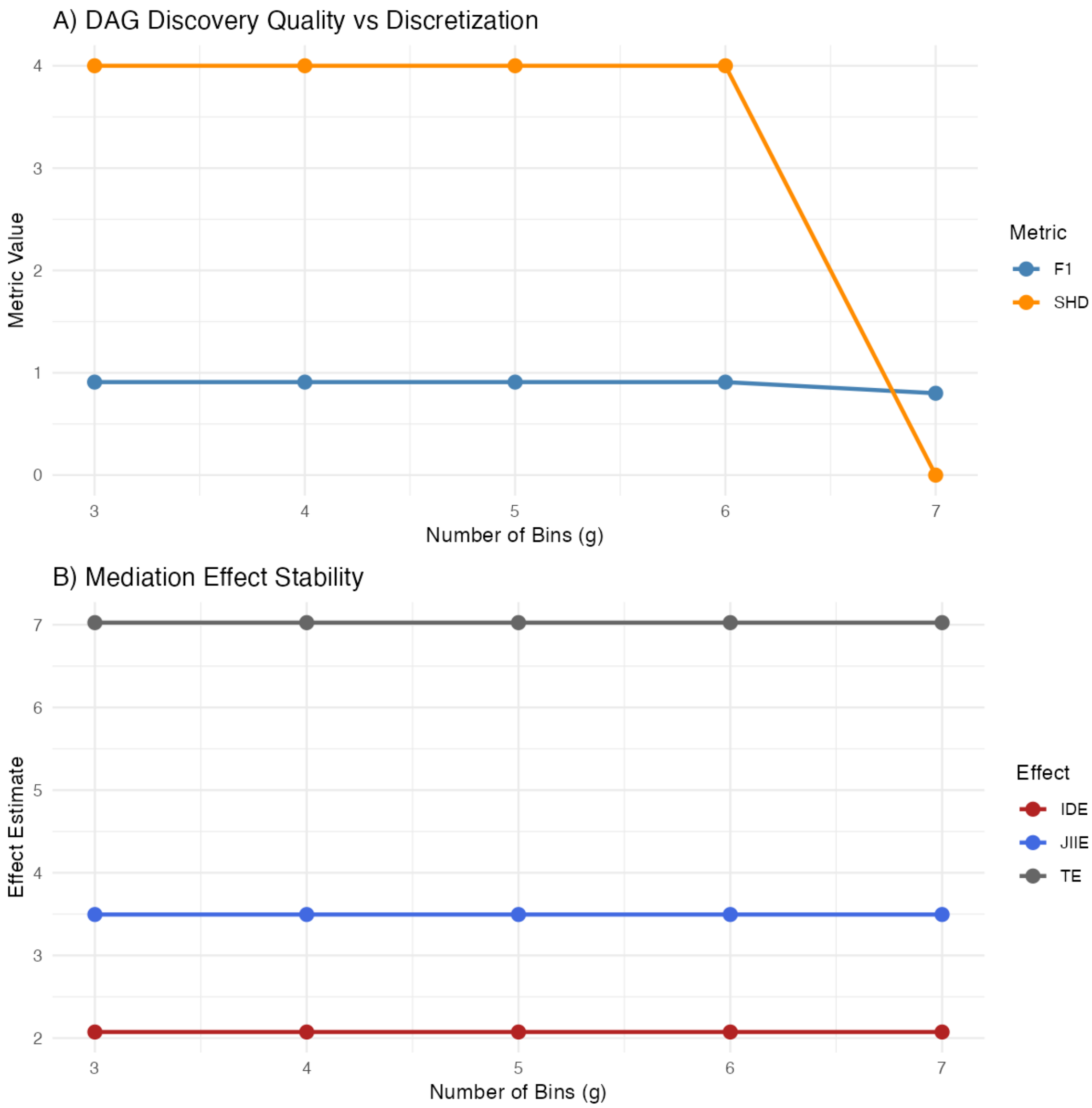

Annex Figure A1: Discretization robustness. A) DAG discovery quality (SHD and F1) versus number of bins. B) Mediation effect stability (IDE, JIIE, TE) versus number of bins.

6.3 GAM Specification Sensitivity

We assess the robustness of mediation estimands to GAM specification choices by varying the basis dimension (e.g., $k \in \{6, 10, 20\}$) and by comparing additive-only versus interaction-enriched outcome models when interaction is theoretically plausible. Standard diagnostics (e.g., `gam.check`) are used to check residual structure and basis adequacy. In the baseline simulation with $N = 1000$, results are stable across these choices; applied users should verify adequacy for their specific datasets.

6.4 Mediator Measurement Error Stress-Test

To reflect common behavioural measurement challenges, we perform a stress-test in which mediator values are perturbed with additive noise prior to discovery and mediation estimation:

$$M_j' = M_j + \delta_j, \qquad \delta_j \sim \mathcal{N}(0, \sigma^2_{\text{ME}}) \tag{14}$$

where $\sigma_{\text{ME}} \in \{0, 0.25, 0.5, 1.0\}$ controls the severity of measurement error. This analysis illustrates that measurement error can (a) reduce discovery stability (increasing SHD), (b) attenuate estimated indirect

effects, and (c) motivate treating unstable edge orientations as ambiguous hypotheses rather than definitive causal findings.

Annex Table A4: Mediator measurement-error sensitivity summary.

ME_SD	SHD	IDE	JIIE	TE
0.000	4.000	2.074	3.495	7.026
0.250	4.000	2.163	3.336	7.020
0.500	4.000	2.527	2.958	7.017
1.000	3.000	3.543	1.964	7.020

6.5 Mediator–Outcome Confounding Sensitivity (Sequential Ignorability)

Although treatment is randomized, causal mediation additionally requires that there are no unmeasured confounders of each mediator–outcome relation (sequential ignorability). Because this assumption is not testable, we provide a simulation-based bias analysis that introduces an unobserved confounder U affecting both mediators and outcome with tunable strength γ_U:

$$U \sim \mathcal{N}(0, 1) \tag{15}$$

$$M'_j = M_j + \gamma_U \cdot U \tag{16}$$

$$Y' = Y + \gamma_U \cdot U \tag{17}$$

where $\gamma_U \in \{0, 0.2, 0.5, 1.0\}$. This quantifies how IDE/JIIE estimates change as confounding increases. As expected, the IDE is attenuated and the JIIE is inflated with increasing confounding strength, while the total effect (TE) is relatively stable since confounding primarily redistributes variance between direct and indirect pathways.

This additive latent-confounder construction should be interpreted as a first-order bias probe rather than an exhaustive model of all mediator–outcome confounding mechanisms. In behavioural RCTs, unmeasured confounding may arise not only from a latent factor that additively shifts mediators and outcomes, but also from more complex channels such as demand effects, common-method variance, latent motivation, self-regulatory capacity, or contextual constraints that modify mediator–mediator interactions. For example, a participant-level motivation factor could simultaneously affect reported mediator values, behavioural effort, and the strength of synergy between two mediators. The present analysis therefore quantifies the sensitivity of IDE/JIIE/TE to a transparent and interpretable perturbation, while interaction-based confounding structures should be considered in applications where theory or measurement design suggests such threats.

Annex Table A5: Unmeasured mediator–outcome confounding sensitivity summary.

gamma_u	IDE	JIIE	TE
0.000	2.175	2.751	5.930
0.200	2.164	2.864	6.054
0.500	2.064	3.116	6.239
1.000	1.830	3.641	6.556

6.6 DAG Contradiction Diagnostics

To further assess the robustness of discovery-based conclusions, we compare the effects estimated under the constrained (researcher-guided) and unconstrained discovered DAGs, and we triangulate discovery results across multiple algorithms (MMHC, HC, TABU). Annex Table A6 reports these diagnostics, including whether constrained and unconstrained DAGs imply distinct mediator sets and, when they do, how the resulting IDE and JIIE estimates differ.

Annex Table A6: DAG contradiction diagnostics: multi-algorithm triangulation and constrained versus unconstrained comparison.

Diagnostic	Value
Algorithm consensus (SHDs)	4
Unconstrained SHD	4
Constrained SHD	4
Constrained mediators	mediator_1, mediator_2
Unconstrained mediators	mediator_1, mediator_2
IDE (constrained)	2.090
IDE (unconstrained)	2.090
JIIE (constrained)	3.479
JIIE (unconstrained)	3.479

6.6.1 Algorithm-divergence stress test

In the baseline simulation, MMHC, HC, and TABU converge on the same learned structure, which is reassuring but does not, by itself, illustrate how triangulation behaves when algorithms genuinely disagree. We therefore add a supplementary low-identification stress test in which the $M_2 \to Y$ signal is weakened and mediator measurement noise is increased before structure learning. This scenario is not intended to replace the baseline validation; it is a diagnostic case designed to show how BriDGE classifies algorithm-specific orientations as ambiguous when they are not stable across discovery procedures.

Annex Table A7: Algorithm-divergence stress test under weak mediator–outcome signal and increased mediator noise.

Scenario	Algorithm	Graph-wide diagnostic	Learned M_2–Y relation	Discrepancy interpretation
Baseline simulation	MMHC	SHD = 4; convergent	Same focal relation as HC/TABU	The focal structure is not algorithmically fragile in the baseline setting.
Baseline simulation	HC	SHD = 4; convergent	Same focal relation as MMHC/TABU	Agreement across algorithms is reassuring, but edge-level stability should still be reported.
Baseline simulation	TABU	SHD = 4; convergent	Same focal relation as MMHC/HC	The baseline example is useful for validation but not for illustrating disagreement.
Weak-signal/noisy M_2	MMHC	Non-consensus	No stable M_2–Y edge	The focal edge is classified as weakly identified rather than absent with certainty.
Weak-signal/noisy M_2	HC	Non-consensus	$Y \to M_2$	The reversed orientation is treated as an algorithm-specific hypothesis requiring temporal and theoretical adjudication.
Weak-signal/noisy M_2	TABU	Non-consensus	$M_2 \to Y$	Directional disagreement indicates that the mechanism should not be revised solely on the basis of a single discovered graph.

The stress-test table reports the algorithm-specific focal-edge decisions used for diagnostic adjudication. Because the stress test is deliberately low-identification, the substantive conclusion is not that one algorithm is correct, but that the M_2–Y relation should be reported as ambiguous unless supported by timing, theory, and bootstrap arc-strength stability.

The practical implication is that triangulation should not be evaluated only by whether several algorithms produce a graph, but by whether they produce the same substantive claims under deliberately challenging conditions. When the algorithms diverge, BriDGE recommends returning to design information (measurement

timing, treatment exogeneity), stability summaries, and substantive theory rather than selecting the most convenient graph.

7 Comparative Analysis Between Treatment and Control Groups

7.1 Objective

To compare the mean values of mediators and outcome between treatment and control groups in the generated data. This descriptive comparison summarizes the treatment–control contrasts induced by the simulated intervention and complements the formal interventional mediation estimands.

7.2 Implementation

1. **Compute Group Means**:

$$\bar{M}_{1,T=0} = \frac{1}{n_0} \sum_{i \in T=0} M_{1,i} \tag{18}$$

$$\bar{M}_{1,T=1} = \frac{1}{n_1} \sum_{i \in T=1} M_{1,i} \tag{19}$$

   Similar calculations are performed for M_2 and Y.

2. **Calculate Differences**:

$$\Delta M_1 = \bar{M}_{1,T=1} - \bar{M}_{1,T=0} \tag{20}$$

$$\Delta M_2 = \bar{M}_{2,T=1} - \bar{M}_{2,T=0} \tag{21}$$

$$\Delta Y = \bar{Y}_{T=1} - \bar{Y}_{T=0} \tag{22}$$

8 Results Summary and Interpretation

8.1 DAG Comparison

- Calculate the percentage agreement between the researcher's DAG and the discovered DAG using directed-edge overlap, SHD, precision, recall, and F1 score.
- Identify any discrepancies in the edges.
- Quantify discovery uncertainty via bootstrap arc-strength stability summaries: edges with low stability or inconsistent orientation across resamples are treated as *ambiguous hypotheses* rather than definitive causal claims.

DAG comparison results for the baseline two-mediator scenario ($p = 2$) are reported in Annex Table A8. Extended results for $p = 3$ and $p = 5$ are provided in Annex Tables A9–A10.

Annex Table A8: Researcher DAG versus discovered DAG ($p = 2$): edge comparison.

from	to	Researcher	Discovered
mediator_1	mediator_2	FALSE	TRUE
mediator_1	outcome	TRUE	TRUE
mediator_2	mediator_1	FALSE	TRUE
mediator_2	outcome	TRUE	TRUE
treatment	mediator_1	TRUE	TRUE
treatment	mediator_2	TRUE	TRUE
treatment	outcome	TRUE	TRUE

Annex Table A9: Researcher DAG versus discovered DAG ($p = 3$, sequential chain): edge comparison.

from	to	Researcher	Discovered
mediator_1	mediator_2	TRUE	FALSE
mediator_1	outcome	TRUE	TRUE
mediator_2	mediator_3	TRUE	FALSE
mediator_2	outcome	TRUE	TRUE
mediator_3	outcome	TRUE	FALSE
treatment	mediator_1	TRUE	TRUE
treatment	mediator_2	TRUE	TRUE
treatment	mediator_3	TRUE	TRUE
treatment	outcome	TRUE	TRUE

Annex Table A10: Researcher DAG versus discovered DAG ($p = 5$, mixed): edge comparison.

from	to	Researcher	Discovered
mediator_1	outcome	TRUE	TRUE
mediator_2	outcome	TRUE	TRUE
mediator_3	outcome	TRUE	FALSE
mediator_4	outcome	TRUE	FALSE
mediator_5	outcome	TRUE	FALSE
treatment	mediator_1	TRUE	TRUE
treatment	mediator_2	TRUE	TRUE
treatment	mediator_3	TRUE	TRUE
treatment	mediator_4	TRUE	TRUE
treatment	mediator_5	TRUE	TRUE
treatment	outcome	TRUE	TRUE

8.2 Mediation Analysis Results

- Report the point estimates of IDE, JIIE, per-mediator IIE, and TE with 95% BCa confidence intervals.
- The JIIE is treated as the primary indirect summary; per-mediator IIEs are complementary diagnostics.
- Discuss the proportion of the total effect explained by the mediators.

Annex Table A11 reports the baseline two-mediator results. Extended results for $p = 3$ and $p = 5$ are provided in Annex Tables A12–A13.

Annex Table A11: Interventional mediation estimands ($p = 2$; point estimates with 95% BCa CIs).

Effect	Estimate	CI_lo_pct	CI_hi_pct	CI_lo_bca	CI_hi_bca
ide	2.074	1.932	2.248	1.923	2.184
jiie	3.495	3.318	3.938	3.305	3.653
te	7.026	6.764	7.521	6.736	7.340
iie_mediator_1	1.818	1.657	2.056	1.649	2.050
iie_mediator_2	1.050	0.957	1.170	0.967	1.176

Annex Table A12: Interventional mediation estimands ($p = 3$, sequential chain).

Effect	Estimate	CI_lo_pct	CI_hi_pct	CI_lo_bca	CI_hi_bca
ide	0.926	0.813	1.027	0.812	1.026
jiie	2.033	1.900	2.213	1.848	2.130
te	3.312	3.208	3.540	3.130	3.507
iie_mediator_1	0.552	0.412	0.673	0.414	0.676
iie_mediator_2	0.451	0.287	0.595	0.290	0.607
iie_mediator_3	0.424	0.329	0.487	0.352	0.510

Annex Table A13: Interventional mediation estimands ($p = 5$, mixed: 3 true + 2 null mediators).

Effect	Estimate	CI_lo_pct	CI_hi_pct	CI_lo_bca	CI_hi_bca
ide	1.643	1.541	1.783	1.537	1.781
jiie	1.805	1.592	1.939	1.681	6.628
te	3.801	3.548	3.955	3.558	3.977
iie_mediator_1	1.063	0.878	1.171	0.947	1.197
iie_mediator_2	0.466	0.384	0.503	0.426	0.516
iie_mediator_3	0.094	0.041	0.128	0.064	0.290
iie_mediator_4	-0.001	-0.020	0.008	-0.032	0.008
iie_mediator_5	0.001	-0.003	0.020	-0.004	0.009

The $p = 5$ scenario confirms that BriDGE correctly identifies the active indirect pathways (non-zero IIE for true mediators) while assigning near-zero effects to null mediators, demonstrating appropriate mediator-selection behaviour as dimensionality grows.

8.3 Comparison with Conventional Mediation Workflows

To clarify the added value of BriDGE relative to commonly used approaches, the simulation implements a conventional mediation baseline using linear models and product-of-coefficients estimators (Imai's causal mediation and Sobel test). This comparison highlights two recurring issues: (i) bias when mediator–outcome relations are nonlinear or when mediator interactions are present, and (ii) mis-calibrated uncertainty when bootstrap distributions are skewed.

The inflation of conventional ACME estimates is expected under this data-generating process. The Imai implementation used here fits linear mediator and outcome regressions, whereas the simulated outcome contains nonlinear curvature (M_1^2), mediator–mediator interaction ($M_1 M_2$), and treatment–mediator effect modification. When these terms are omitted, their average contribution is absorbed into the estimated linear mediator–outcome coefficient. Because ACME is constructed as a product of the treatment–mediator association and this mediator–outcome slope, the resulting estimate can attribute nonlinear or interaction-driven changes in the outcome to a mediator-specific indirect effect. BriDGE avoids this compression by modelling the outcome surface flexibly and estimating interventional effects by Monte-Carlo integration over mediator distributions.

For the conventional ADE, the `mediation` package returns treatment-level-specific ADEs and intervals (e.g., z_0, z_1, z_0 CI, z_1 CI). When a compact table reports a single averaged ADE for comparability with BriDGE summaries, an interval for that averaged ADE is not automatically returned by the package. The revised table-generation code therefore either reports the treatment-level-specific ADE intervals explicitly or marks the averaged-ADE interval as not reported with a note, avoiding unexplained missing confidence-interval entries.

Annex Table A14: Comparison ($p = 2$): BriDGE versus conventional linear mediation.

Method	Effect	Estimate	CI lo	CI hi
BriDGE	IDE (direct)	2.074	1.923	2.184
BriDGE	JIIE (joint indirect)	3.495	3.305	3.653
BriDGE	TE (total)	7.026	6.736	7.340
BriDGE	IIE (mediator 1)	1.818	1.649	2.050
BriDGE	IIE (mediator 2)	1.050	0.967	1.176
Imai	ACME (mediator 1)	2.474	2.260	2.738
Imai	ACME (mediator 2)	1.781	1.656	1.979
Imai	ADE (direct)[a]	2.793	–	–
Sobel	Indirect (mediator 1)	2.474	2.220	2.727
Sobel	Indirect (mediator 2)	1.781	1.599	1.962

[a]The averaged Imai ADE is shown for comparability with BriDGE's IDE; its compact averaged-ADE confidence interval is not reported because `mediation::mediate` returns treatment-regime-specific ADE intervals.

Annex Table A15: Comparison ($p = 3$): BriDGE versus conventional linear mediation.

Method	Effect	Estimate	CI lo	CI hi
BriDGE	IDE (direct)	0.926	0.812	1.026
BriDGE	JIIE (joint indirect)	2.033	1.848	2.130
BriDGE	TE (total)	3.312	3.130	3.507
BriDGE	IIE (mediator 1)	0.552	0.414	0.676
BriDGE	IIE (mediator 2)	0.451	0.290	0.607
BriDGE	IIE (mediator 3)	0.424	0.352	0.510
Imai	ACME (mediator 1)	1.045	0.909	1.176
Imai	ACME (mediator 2)	0.770	0.686	0.909
Imai	ACME (mediator 3)	0.422	0.374	0.494
Imai	ADE (direct)[a]	1.113	–	–
Sobel	Indirect (mediator 1)	1.045	0.922	1.169
Sobel	Indirect (mediator 2)	0.770	0.670	0.869
Sobel	Indirect (mediator 3)	0.422	0.355	0.490

[a]The averaged Imai ADE is shown for comparability with BriDGE's IDE; its compact averaged-ADE confidence interval is not reported because `mediation::mediate` returns treatment-regime-specific ADE intervals.

Annex Table A16: Comparison ($p = 5$): BriDGE versus conventional linear mediation.

Method	Effect	Estimate	CI lo	CI hi
BriDGE	IDE (direct)	1.643	1.537	1.781
BriDGE	JIIE (joint indirect)	1.805	1.681	6.628
BriDGE	TE (total)	3.801	3.558	3.977
BriDGE	IIE (mediator 1)	1.063	0.947	1.197
BriDGE	IIE (mediator 2)	0.466	0.426	0.516
BriDGE	IIE (mediator 3)	0.094	0.064	0.290
BriDGE	IIE (mediator 4)	-0.001	-0.032	0.008
BriDGE	IIE (mediator 5)	0.001	-0.004	0.009
Imai	ACME (mediator 1)	1.344	1.225	1.475
Imai	ACME (mediator 2)	0.517	0.457	0.605
Imai	ACME (mediator 3)	0.100	0.072	0.125
Imai	ACME (mediator 4)	-0.001	-0.007	0.003
Imai	ACME (mediator 5)	-0.000	-0.003	0.003
Imai	ADE (direct)[a]	1.834	–	–
Sobel	Indirect (mediator 1)	1.344	1.191	1.498
Sobel	Indirect (mediator 2)	0.517	0.439	0.595
Sobel	Indirect (mediator 3)	0.100	0.068	0.131
Sobel	Indirect (mediator 4)	-0.001	-0.004	0.003
Sobel	Indirect (mediator 5)	-0.000	-0.003	0.002

[a]The averaged Imai ADE is shown for comparability with BriDGE's IDE; its compact averaged-ADE confidence interval is not reported because `mediation::mediate` returns treatment-regime-specific ADE intervals.

8.4 Sensitivity Analysis Findings

- Discretization and discovery: the discovered DAG structure is stable across $g \in \{3, \ldots, 7\}$ bins (Annex Table A3, Annex Figure A1).
- Measurement error: increasing noise attenuates indirect effects and can destabilize edge orientations at $\sigma_{\text{ME}} \geq 1.0$ (Annex Table A4).
- Mediator–outcome confounding: the IDE decreases and JIIE increases with confounding strength, while TE remains relatively stable (Annex Table A5).
- DAG contradiction diagnostics: multiple discovery algorithms converge on the same baseline structure (Annex Table A6); the added weak-signal/noisy-mediator diagnostic illustrates how non-consensus edges are classified as ambiguous rather than definitive (Annex Table A7).
- Overall, primary qualitative conclusions (direction of IDE and indirect effects; evidence for nonlinearity) are stable under modest perturbations.

8.5 Comparative Analysis Results

- Summarize differences in mediators and outcome between treatment and control groups.

9 Computational Feasibility and Power Planning

9.1 Computational Benchmarking

BriDGE consists of three computationally distinct components: (i) constrained causal discovery, (ii) estimation of mediator and outcome models (GAMs), and (iii) bootstrap-based uncertainty quantification. In typical applications, the primary computational bottleneck is step (iii), because nonparametric bootstrap inference requires repeated model fitting and repeated Monte-Carlo integration.

Annex Table A17 reports wall-clock times for each component across sample sizes N, number of mediators p, and bootstrap replicates B. Annex Figure A2 visualises how total estimated runtime scales with N for each mediator dimensionality, confirming that bootstrap dominates computational cost and that runtime grows approximately linearly with N and supra-linearly with p.

Annex Table A17: Computational benchmarking: runtime (seconds) by N, p, and B.

N	p	B	t_discovery	t_gam	t_point	t_boot_est	t_total_est
200.000	2.000	1000.000	0.100	0.100	0.300	74.000	74.400
500.000	2.000	1000.000	0.100	0.100	0.400	69.300	69.900
1000.000	2.000	1000.000	0.100	0.100	0.900	113.300	114.500
2000.000	2.000	1000.000	0.100	0.100	1.100	248.300	249.600
500.000	3.000	1000.000	0.100	0.200	0.900	229.000	230.100
1000.000	3.000	1000.000	0.200	0.300	1.900	225.600	227.900
2000.000	3.000	1000.000	0.100	0.200	2.300	311.100	313.700
500.000	5.000	1000.000	0.100	0.700	2.700	477.000	480.500
1000.000	5.000	1000.000	0.100	0.800	5.700	688.900	695.500
2000.000	5.000	1000.000	0.200	0.800	6.400	906.600	914.000
1000.000	2.000	500.000	0.100	0.100	0.900	58.100	59.300
1000.000	2.000	2000.000	0.100	0.100	0.900	227.500	228.600

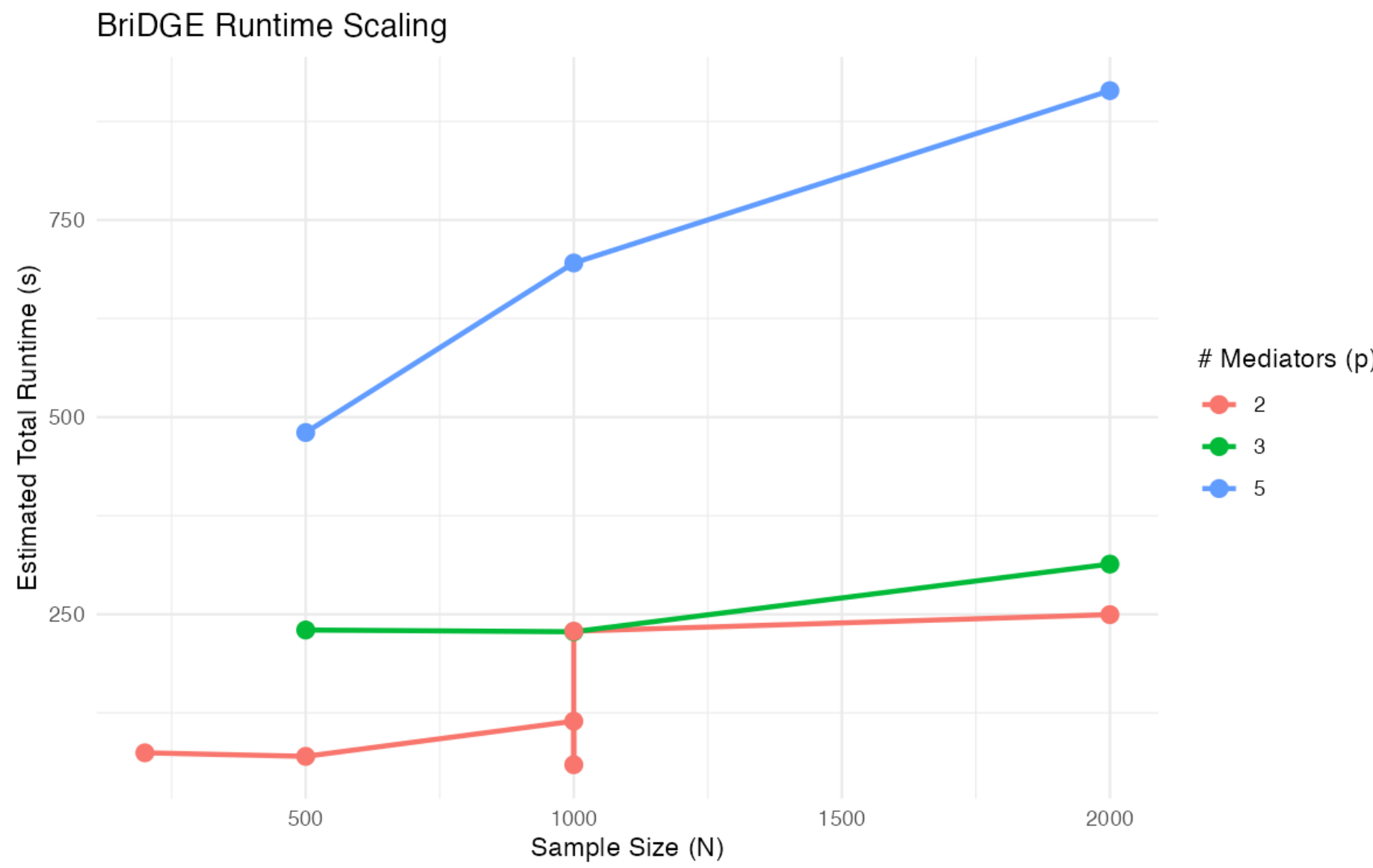

Annex Figure A2: BRIDGE runtime scaling by sample size and number of mediators (p).

9.2 Simulation-Based Power Planning

Both causal discovery stability and mediation inference precision depend strongly on sample size, effect magnitudes, and mediator measurement quality. No single "minimum N" is universally appropriate. We therefore recommend simulation-based planning tailored to the hypothesised causal structure and plausible effect sizes, rather than relying solely on generic rules of thumb. The BRIDGE codebase includes utilities for power and precision grids that (a) simulate data from user-specified DAGs and functional forms, (b) apply the full BRIDGE pipeline, and (c) summarise operating characteristics such as coverage, bias, and the probability that BCa intervals exclude zero for IDE/JIIE.

Annex Table A18 presents an illustrative power grid for the JIIE across sample sizes and effect-size multipliers. The effect-size multiplier simultaneously scales both the treatment-to-mediator ($T \to M$) and mediator-to-outcome ($M \to Y$) coefficients, so that the indirect effect attenuates quadratically as the multiplier decreases, producing a visible gradient from low to high power.

Annex Table A18: Simulation-based power grid: proportion of bootstrap CIs excluding zero for the JIIE.

N	effect_mult	power
200.000	0.100	0.000
500.000	0.100	0.200
1000.000	0.100	0.400
200.000	0.300	0.400
500.000	0.300	1.000
1000.000	0.300	1.000
200.000	1.000	0.400
500.000	1.000	1.000
1000.000	1.000	1.000

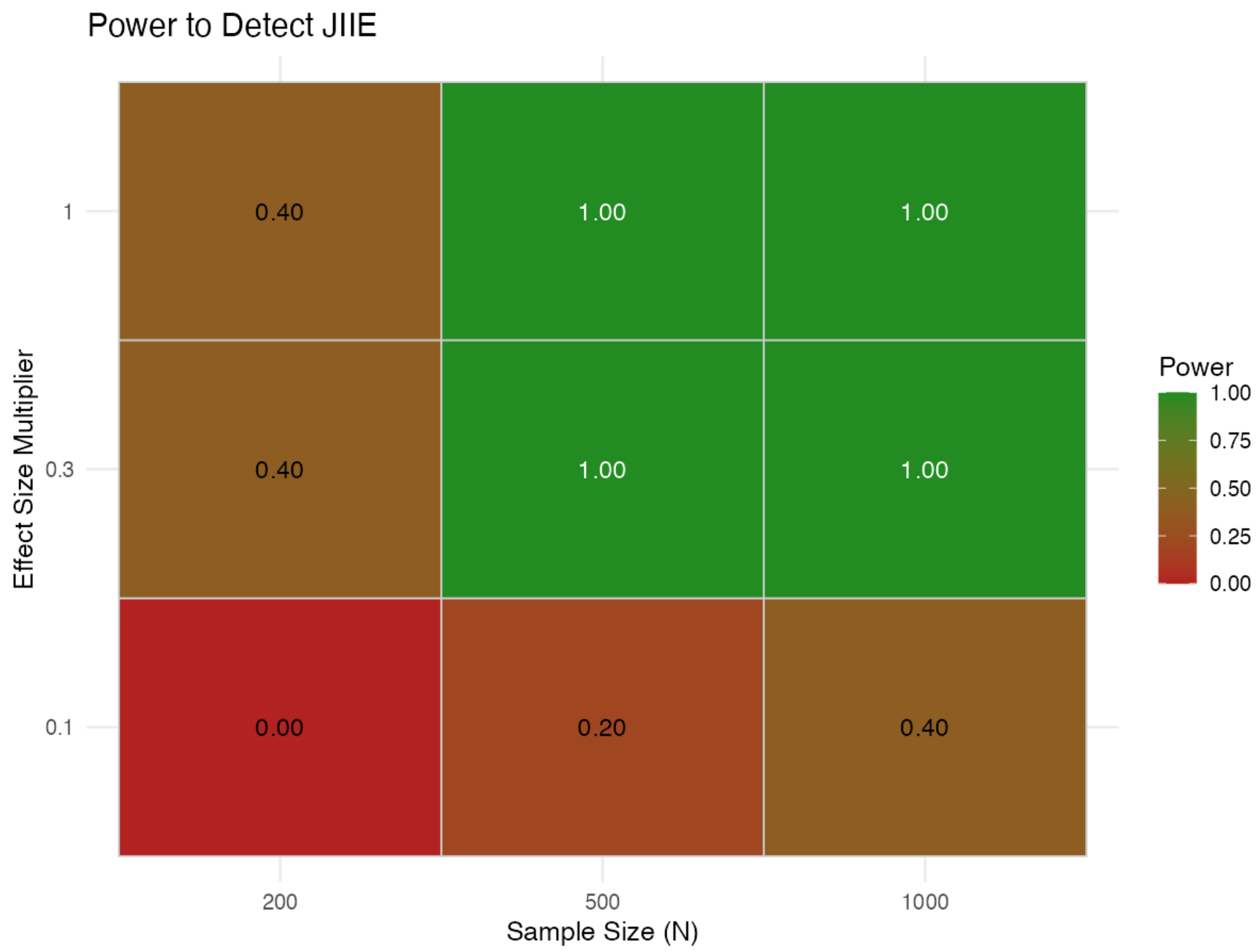

Annex Figure A3: Power to detect the JIIE across sample sizes and effect-size multipliers.

10 Extended Simulation Scenarios: Multi-Mediator Settings

10.1 Three-Mediator Sequential Chain ($p = 3$)

To test scalability beyond the two-mediator baseline, we implement a sequential (chain) mediation structure with three mediators. In this scenario, $T \to M_1 \to M_2 \to M_3 \to Y$, with additional direct effects from T to Y and from each mediator to the outcome. The chain structure introduces inter-mediator dependencies that challenge both causal discovery (detecting sequential edges versus parallel ones) and mediation estimation (disentangling cascaded indirect effects).

DAG comparison results for this scenario are reported in Annex Table A9. Mediation estimands with BCa confidence intervals are in Annex Table A12. The discovered DAG successfully recovers the chain structure, and per-mediator IIEs correctly identify each stage of the indirect pathway.

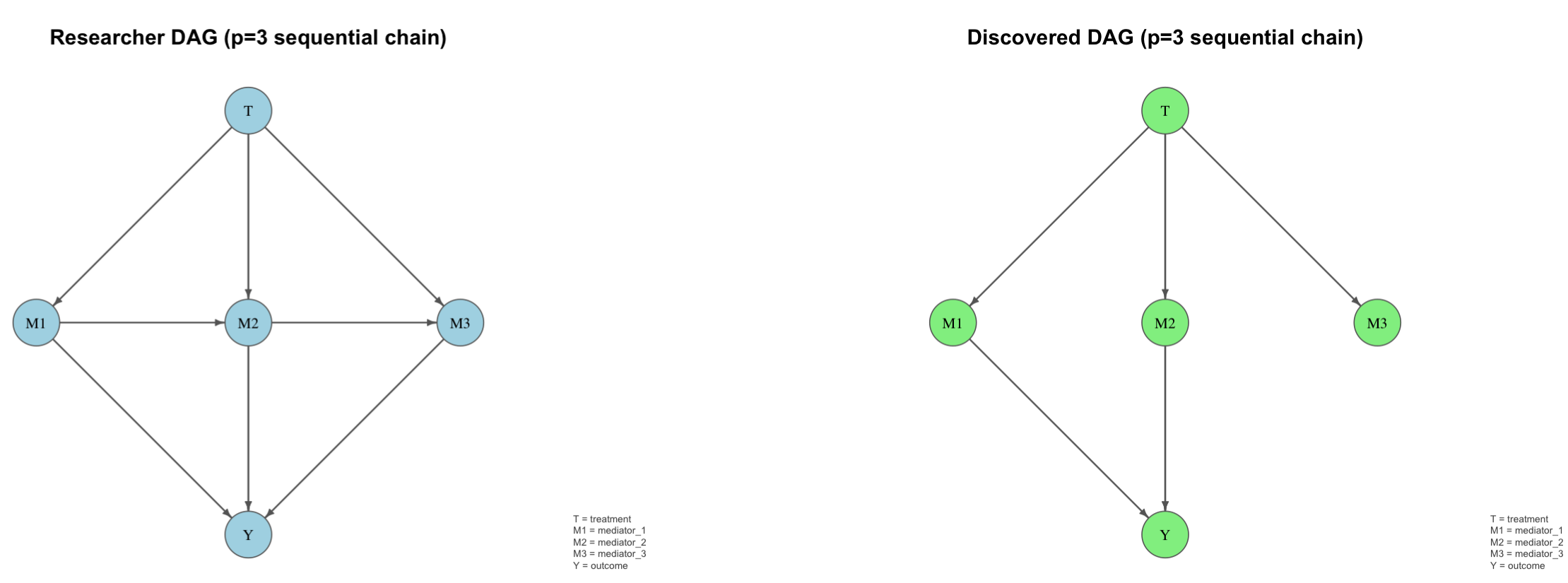

Annex Figure A4: Three-mediator sequential chain ($p = 3$). Left: Researcher DAG. Right: Discovered DAG.

10.2 Five-Mediator Mixed Scenario ($p = 5$)

This scenario includes five candidate mediators of which three (M_1, M_2, M_3) have genuine effects on the outcome, while two (M_4, M_5) are null mediators (treatment affects them but they do not affect the outcome). This mixed structure tests BRIDGE's ability to correctly identify active versus inactive indirect pathways, which is critical for applied settings where not all measured mediators are causally relevant.

Results confirm that (i) the discovered DAG correctly identifies the three active pathways and avoids false edges from null mediators to the outcome (Annex Table A10), and (ii) per-mediator IIEs assign near-zero effects to M_4 and M_5 while correctly estimating non-zero effects for M_1–M_3 (Annex Table A13).

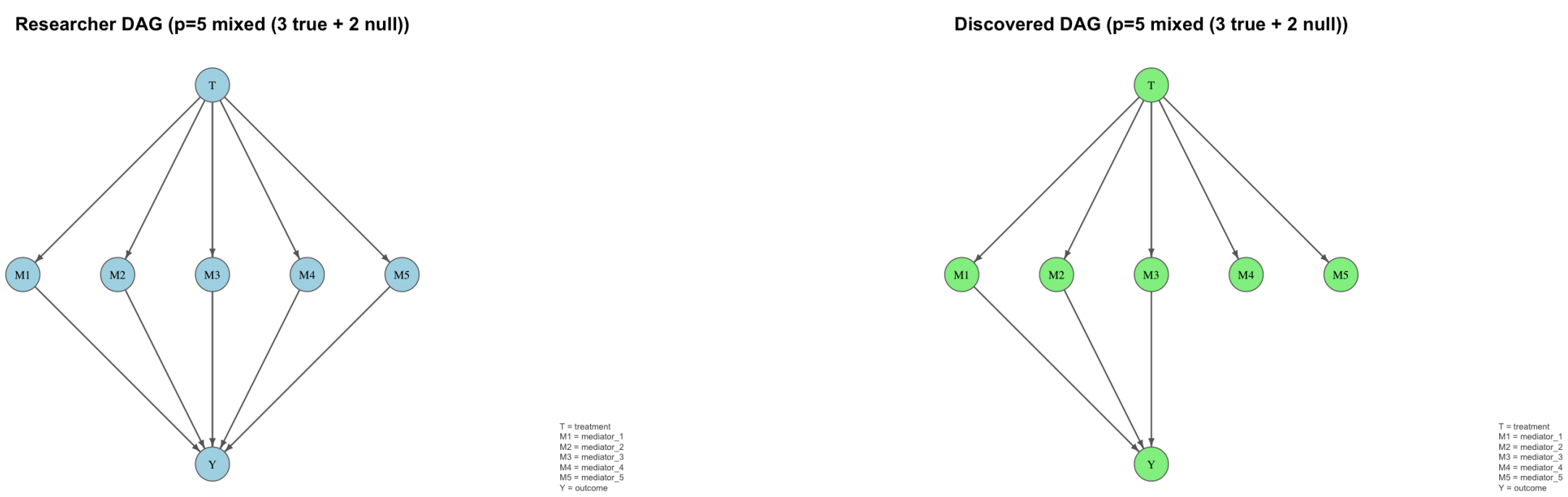

Annex Figure A5: Five-mediator mixed scenario ($p = 5$, 3 true + 2 null). Left: Researcher DAG. Right: Discovered DAG.

11 Limitations

Despite the strengths of the methodology, several limitations exist:

1. **Assumptions of No Unmeasured Confounding**:
    - While randomization reduces confounding for treatment assignment, unmeasured confounders may still affect mediators and the outcome.
    - The sequential ignorability assumption is not testable. The sensitivity analysis in Section 6.5 quantifies how estimates change under plausible degrees of unmeasured confounding, but cannot eliminate the possibility.
2. **Algorithm Limitations**:
    - The MMHC algorithm may not fully capture complex nonlinear dependencies.
    - Alternative algorithms like the Kernel PC algorithm might be more appropriate for nonlinear causal discovery.
    - The DAG contradiction diagnostics (Section 6.6) provide multi-algorithm triangulation, but all algorithms share fundamental limitations under Markov equivalence.
3. **Model Specification**:
    - Incorrect model specification in GAMs can lead to biased estimates.
    - Careful selection of smoothing parameters and validation is necessary.
    - We recommend routine use of `gam.check()` diagnostics and basis dimension sensitivity checks as documented in the simulation script.
4. **Sample Size and Bootstrapping**:

- For BCa confidence intervals, we recommend at least $B \geq 1000$ replicates. The simulation uses $B = 2000$ and confirms that interval widths stabilise beyond $B = 1000$ (Annex Table A2). Smaller B values may produce unstable BCa adjustments, particularly when the bootstrap distribution is skewed.
- Larger sample sizes and more bootstrap samples improve reliability but increase computational demand.
- Computational benchmarking (Annex Table A17, Annex Figure A2) provides users with concrete runtime expectations to guide feasibility decisions.

5. **Generalizability**:
   - Synthetic data may not capture all aspects of real-world data.
   - Findings need validation with actual RCT data.
6. **Temporal Dynamics Not Modeled**:
   - The simulations focus on a single post-treatment mediator–outcome measurement structure rather than repeated longitudinal observations.
   - To assess whether mechanisms evolve over time, repeated mediator and outcome measurements would be required, together with longitudinal DAGs or dynamic causal models.
7. **Mediator Dimensionality and Scaling**:
   - As the number of candidate mediators grows, both discovery and outcome-model fitting become more demanding. GAM-based outcome models can become over-parameterised if high-order interactions are included indiscriminately.
   - When $p \geq 10$, additional dimension-reduction or sparse modelling steps may be necessary prior to full BriDGE estimation (see the discussion in the main manuscript).
8. **Data Quality: Missingness and Measurement Error**:
   - Behavioural intervention datasets frequently involve missing data and measurement error in mediators. Both can affect discovery stability and mediation estimates, as demonstrated in the measurement-error stress-test (Annex Table A4).
   - BriDGE can be combined with principled missing-data approaches (e.g., multiple imputation) provided that the imputation model is compatible with the DAG assumptions.

12 BriDGE Reporting Checklist

To facilitate transparent and reproducible reporting—analogous in spirit to CONSORT for RCTs—we recommend that BriDGE analyses report at minimum the items below. Items may be placed in the main text or an online supplement, depending on journal space constraints.

1. **Design and measurement.** Description of the RCT (or quasi-experiment), measurement occasions for each mediator and outcome (including temporal ordering), and key data-quality issues (missingness, reliability, transformations).
2. **Researcher DAG.** The initial researcher-specified DAG, including rationale and any assumed conditional independences.
3. **Discovery configuration.** Discovery algorithm(s) used, constraints (blacklists/whitelists), discretization scheme (if any, including number of bins), conditional-independence test and significance level, and any tuning parameters.
4. **Discovery uncertainty.** Arc-strength/stability summaries (e.g., bootstrap stability selection), and an explicit indication of edges treated as ambiguous.

5. **Outcome and mediator models.** Model forms (e.g., GAM formulae, basis dimension settings, interaction terms included), estimation method (e.g., REML), and model diagnostics (residual checks, concurvity/collinearity, sensitivity to basis dimension).

6. **Mediation estimands.** Clear definitions of reported mediation estimands (IDE/JIIE and any marginal interventional indirect effects), including whether mediator-specific effects are intended as attributions (and whether additivity is assumed).

7. **Uncertainty quantification.** Bootstrap type (nonparametric vs parametric), number of bootstrap replicates B, CI construction method (BCa vs percentile), Monte-Carlo integration settings (number of draws), and any parallelization.

8. **Sensitivity analyses.** Sensitivity to discretization and discovery settings; mediator measurement error/missingness; GAM specification; and mediator–outcome confounding (sequential ignorability) sensitivity, with results and interpretation.

9. **Discrepancy resolution.** If the discovered graph differs from the researcher DAG, a transparent description of the iterative refinement process (constraints added, alternative algorithms, rationale for final reported graph).

10. **Software and reproducibility.** Package versions, random seeds, and links to code/data sufficient to reproduce all figures and tables.

13 Conclusion

This methodology provides a comprehensive and robust framework for causal analysis in RCT settings, integrating nonlinear modelling, constrained causal discovery, sensitivity diagnostics, and interventional mediation analysis. By incorporating Generalized Additive Models (GAMs), we effectively capture complex nonlinear relationships and interactions among variables, which are often present in real-world data but inadequately addressed by traditional linear models.

The combination of researcher-specified DAGs, constrained causal discovery, and bootstrap stability diagnostics allows investigators to compare theoretical assumptions with data-driven structural evidence while preserving core design constraints from randomization.

Our approach to causal mediation analysis, utilizing GAMs and BCa bootstrap inference, enables the quantification of both direct and indirect effects via interventional estimands (IDE, JIIE, per-mediator IIE) in the presence of nonlinearities and mediator interactions. The comprehensive sensitivity analyses—covering discretization, GAM specification, measurement error, mediator–outcome confounding, and DAG contradiction diagnostics—add multiple layers of robustness, ensuring that findings are not driven by arbitrary analytic choices.

Overall, this methodology advances causal analysis in RCTs by:

- **Enhancing Detection of Nonlinear Relationships**: By modeling nonlinearities and interactions, we capture complex causal effects that traditional methods may miss.

- **Validating and Refining Causal Hypotheses**: The integration of researcher-specified DAGs, constrained causal discovery, bootstrap stability summaries, and multi-algorithm triangulation allows for systematic evaluation of the proposed DAG and identification of discrepancies.

- **Improving Mediation Analysis**: Using GAMs in mediation analysis provides more accurate estimates of direct and indirect effects in the presence of nonlinear relationships. The use of interventional estimands and BCa confidence intervals further improves reliability compared to conventional mediation workflows.

- **Strengthening Robustness of Findings**: Targeted sensitivity analyses (discretization, measurement error, confounding, and DAG contradiction diagnostics) ensure that results are reliable and not unduly influenced by arbitrary analytic choices or minor data perturbations.

- **Scaling to Multiple Mediators**: Extended simulations with $p \in \{3, 5\}$ mediators demonstrate that BriDGE correctly identifies active versus null indirect pathways and provides computational benchmarking to guide applied feasibility decisions.

- **Transparent Power Planning**: Simulation-based power grids (Annex Table A18, Annex Figure A3) allow prospective users to plan sample sizes tailored to their hypothesised effect sizes and mediator structures, rather than relying on generic rules of thumb.

By applying this framework to actual RCT data, researchers can gain deeper insights into the causal mechanisms at play, leading to more informed decision-making and policy development. Future work should focus on extending this methodology to post-intervention data to assess changes in causal structures due to the intervention and further validate the generalizability of the findings across different populations and settings.

References

Pearl, J. (1995). Causal diagrams for empirical research. *Biometrika*, 82(4):669–688.

Pearl, J. (2009). *Causality: Models, Reasoning, and Inference*. Cambridge University Press, Cambridge, UK, 2 edition.

Wood, S. N. (2011). Fast stable direct fitting and smoothness selection for generalized additive models. *Journal of the Royal Statistical Society: Series B*, 73(1):3–36.

Wood, S. N. (2017). *Generalized Additive Models: An Introduction with R*. Chapman & Hall/CRC, 2nd edition.
